# A Smart Single‐Loop‐Mediated Isothermal Amplification Facilitates Flexible SNP Probe Design for On‐Site Rapid Differentiation of SARS‐CoV‐2 Omicron Variants

**DOI:** 10.1002/advs.202502708

**Published:** 2025-04-01

**Authors:** Qijie Lin, Hongchao Gou, Xiaoyun Qu, Kaiyuan Jia, Yuhui Deng, Dan Li, Qianyi Cai, Yucen Liang, Xiaozhen Xu, Yanbin Li, Jianhan Lin, Letian Li, Yuhang Jiang, Shouwen Du, Lingcong Deng, Bailing Yan, Ruidong Liu, Chang Li, Jianmin Zhang, Ming Liao

**Affiliations:** ^1^ National and Regional Joint Engineering Laboratory for Medicament of Zoonoses Prevention and Control Key Laboratory of Zoonoses Ministry of Agriculture Key Laboratory of Zoonoses Prevention and Control of Guangdong Province Animal Infectious Diseases Laboratory College of Veterinary Medicine South China Agricultural University Guangzhou 510642 China; ^2^ Guangdong Provincial Key Laboratory of Livestock Disease Prevention Scientific Observation and Experiment Station of Veterinary Drugs and Diagnostic Techniques of Guangdong Province Institute of Animal Health Guangdong Academy of Agricultural Sciences Guangzhou 510640 China; ^3^ Key Laboratory of Agricultural Information Acquisition Technology Ministry of Agriculture and Rural Affairs China Agricultural University Beijing 100083 China; ^4^ Department of Respiratory Medicine Center for Infectious Diseases and Pathogen Biology Key Laboratory of Organ Regeneration and Transplantation of the Ministry of Education State Key Laboratory for Zoonotic Diseases The First Hospital of Jilin University Changchun 130021 China; ^5^ Department of Biological and Agricultural Engineering Center of Excellence for Poultry Science University of Arkansas Fayetteville AR 72701 USA; ^6^ Changchun Veterinary Research Institute Research Unit of Key Technologies for Prevention and Control of Virus Zoonoses Chinese Academy of Medical Sciences Changchun 130062 China; ^7^ College of Animal Science and Technology Zhongkai University of Agriculture and Engineering Guangzhou 510225 China

**Keywords:** nucleic acid amplification, SARS‐CoV‐2 variants of concern, SNP detection, ssLAMP

## Abstract

Rapid on‐site typing methods for SARS‐CoV‐2 variants of concern are crucial for its effective surveillance and control. Herein, a smart single‐loop‐mediated isothermal amplification (ssLAMP) method with the absence of an inner primer but the addition of a swarm primer for differentiation of SARS‐CoV‐2 Omicron variants is developed. This unique primer design strategy offers greater flexibility in introducing single nucleotide polymorphism (SNP) identification probes and enables multiple detection assays for SARS‐CoV‐2 Omicron variants including BA.1, BA.2, BA.3, BA.4, and BA.5. A 3D‐printed portable dual fluorescence visualization device and smartphone app are developed to enable point‐of‐care testing. This assay is rapid (within 90 min), highly sensitive (100 copies/reaction), and specific (identification of SNP) for SARA‐CoV‐2 Omicron variants. The ssLAMP method identifies five BA.5‐positive samples among 97 nasopharyngeal swab samples from the clinic, with a 100% concordance rate with Sanger sequencing. The ssLAMP assay system is expected to be utilized for on‐site, highly specific, and rapid visualization detection of SARS‐CoV‐2 and its variants, with great application potential in pathogen genotyping, early cancer screening, and other areas of SNP mutation detection.

## Graphical abstract

The ssLAMP method amplifies the nucleic acid of pathogen and identifies SNP mutations. By combining the portable detection device with smartphone‐based analysis software, enables rapid, on‐site SNP detection and genotyping of SARS‐Cov‐2 Omicron variants, providing a cost‐effective and field‐deployable solution for precise pathogen identification in clinical and resource‐limited settings.

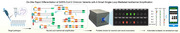

## 1 Introduction

Single nucleotide polymorphism (SNP) is associated with the risk of various diseases including cancer, genetic disorders, and infectious diseases.^[^ ^1^ ^]^ Therefore, SNPs are considered pivotal biomarkers for molecular diagnostics,^[^ ^2^ ^]^ particularly for pathogens with high mutation rates that alter infectivity, transmissibility, and antigenicity. As a representative pathogen, the rapid spread of severe acute respiratory syndrome coronavirus 2 (SARS‐CoV‐2) and its variants caused the COVID‐19 pandemic, posing a significant threat worldwide.^[^ ^3^ ^]^ Currently, its pathogenicity and transmissibility remain difficult to predict due to frequent SNP mutations.^[^ ^4^ ^]^ However, most current methods for SNP detection are laboratory‐dependent and time‐consuming, hindering rapid response and precise prevention and control of SARS‐CoV‐2. Therefore, the development of point‐of‐care testing (POCT) technologies for the rapid and multiplexed detection of SARS‐CoV‐2 mutations is urgently needed.^[^ ^5^ ^]^

Sequencing technologies are considered the gold standard for SNP detection. At present, two principal categories of sequencing technologies are in use: next‐generation sequencing (NGS) and Sanger sequencing combined with reverse transcription‐polymerase chain reaction (RT‐PCR). NGS is the most widely used method for detecting both known and unknown pathogen sequences. It can obtain the complete genome sequence of SARS‐CoV‐2 and accurately analyze all known and unknown SNPs. However, it is a time‐consuming process, typically requiring more than 3 days. It also necessitates sophisticated, expensive sequencing instruments and specialized bioinformatics techniques for data analysis. These limitations significantly restrict its applicability in POCT. Combining Sanger sequencing with RT‐PCR reduces the time required for detection. However, this approach also requires the use of specialized sequencing instruments and trained operators, limiting its use in POCT. To achieve rapid SNP identification, hybridization probes or PCR methods based on enzymatic activation probes were developed to differentiate wild‐type and mutant alleles.^[^ ^6^ ^]^ Compared to PCR, isothermal nucleic acid amplification methods eliminate the need for thermocycling, providing a more convenient diagnostic option. Hence, combining allele‐specific probes with isothermal amplification represents a promising approach for POCT of SNPs.^[^ ^7^ ^]^

Loop‐mediated isothermal amplification (LAMP) is a single‐enzyme‐driven amplification method that is widely used.^[^ ^8^ ^]^ However, its primer design presents challenges for SNP identification. Typical LAMP reactions are initiated by a pair of inner and outer primers to produce double‐looped structures, with the results indicated by nucleic acid dyes or pH indicators.^[^ ^9^ ^]^ However, such dyes or indicators are often linked to the general LAMP product, making them difficult to apply in SNP identification. To address these issues, loop primers^[^ ^10^ ^]^ of LAMP were modified as fluorescent probes. However, such loop primers should be located in the same region as the SNP site. Though LAMP possesses high specificity due to its six primers targeting eight specific regions on the target DNA, its drawback is that the remaining regions were too short and usually not suitable for SNP‐targeted probe design.^[^ ^11^ ^]^ Additionally, the complex primer set of LAMP greatly increases the difficulty of performing multiple target detection in a single reaction. Recently, some POCT technologies based on LAMP have been designed to identify SARS‐CoV‐2 mutations.^[^ ^12^ ^]^ However, most of these methods were used for single target detection, making multiple mutation detection challenging to achieve. Therefore, an advanced isothermal amplification method flexibly combined with allele specific probe is highly desirable.

In this study, we developed a smart single‐loop‐mediated isothermal amplification (ssLAMP) method combined with Rnase H2 enzyme‐activated probe for SNP detection. The ssLAMP exhibited comparable reaction efficiency with conventional LAMP when detecting nucleic acids with both high and low GC content. Using SARS‐CoV‐2 Omicron variants as models, we demonstrated the superior primer design flexibility and SNP detection capability of ssLAMP compared to conventional LAMP. Based on this, we established a multiplex ssLAMP combined with an Rnase H2 enzyme‐activated probe to rapidly differentiate Omicron variants. These variants have diverged into at least five subvariants (BA.1, BA.2, BA.3, BA.4, and BA.5) with emerging sub‐lineages. To realize its POCT application, a dual‐fluorescence visualization device and intelligent analysis software were developed and integrated. By testing 97 clinical samples, its potential application value was evaluated. The ssLAMP method achieved precise detection of SNP mutations, exhibiting comparable performance to the Sanger sequencing in conjunction with RT‐PCR. According to our knowledge, this is the first reported multiplex POCT method for SARS‐CoV‐2 Omicron variants (BA.1, BA.2, BA.3, BA.4, and BA.5). The innovative primer design strategy of the ssLAMP method offers a promising approach for on‐site detection of multiple SNPs in a single‐tube system. This technology has the potential to be applied in a range of fields, including early cancer screening, the detection of drug‐resistant mutations, and the precise genotyping of pathogens.

## 2 Results

### 2.1 Establishment and Proof of Concept of the ssLAMP Reaction System

**Figure 1:**
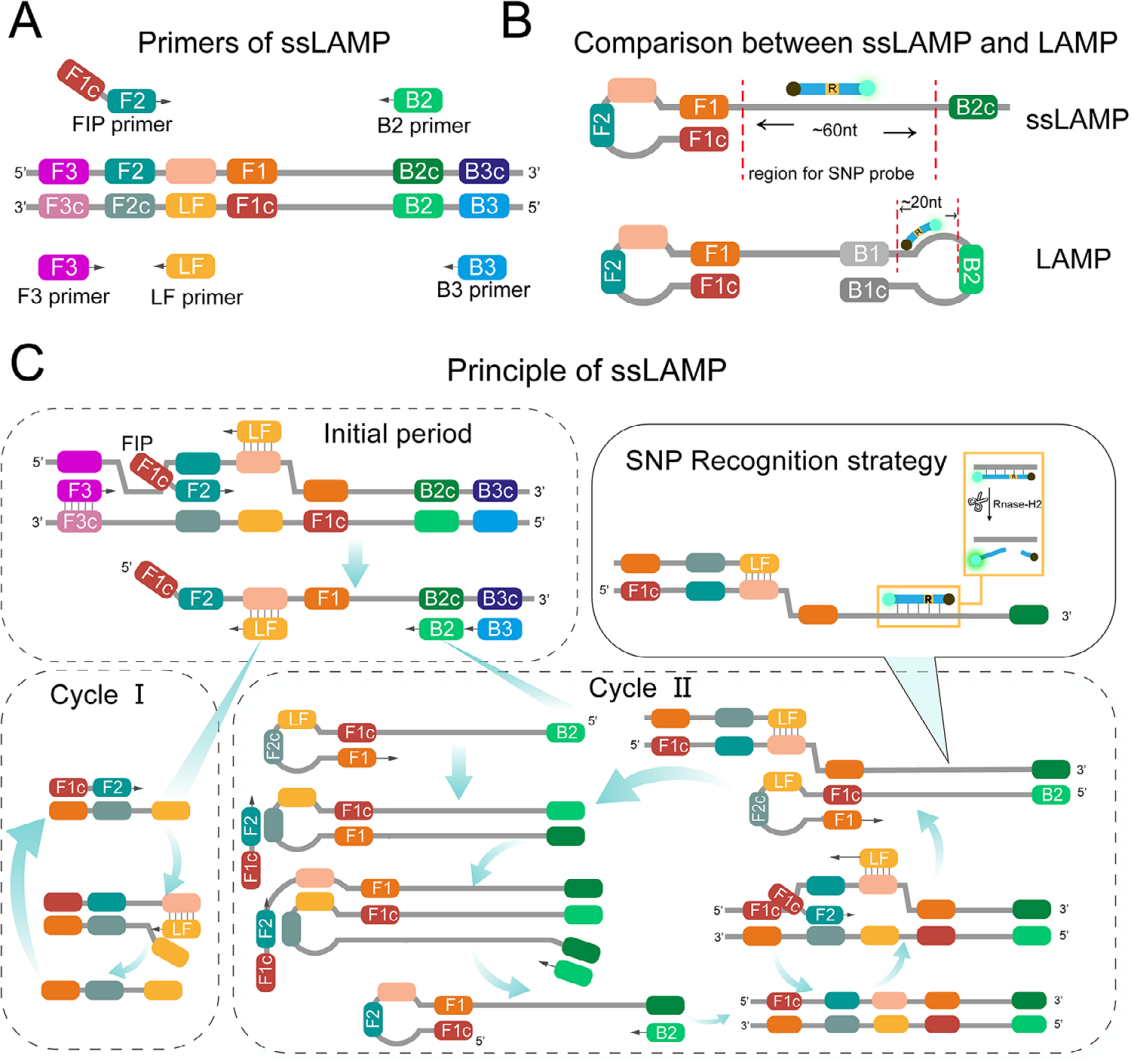
The principle and workflow of ssLAMP method. A) The composition of the ssLAMP primer set. B) The comparison between ssLAMP and LAMP. The ssLAMP has a larger SNP probe configuration area than LAMP C. In the initial period, FIP, aided by F3 and LF, invades the DNA double strand, initiates the ssLAMP reaction, and generates the essential single‐loop product that initiates the cyclic reaction. In the cycling phase, some of the products generated by the ssLAMP reaction will be employed as substrates for the subsequent round of reaction, thereby facilitating exponential amplification of the ssLAMP reaction. In the ssLAMP reaction, the identification of single‐nucleotide polymorphisms (SNPs) can be achieved by introducing an SNP detection probe. When the ribonucleotide base in the SNP probe aligns with the template base, it will be cleaved by Rnase H2, resulting in the generation of a fluorescent signal.

The LAMP technique faces several challenges, particularly in probe design. One major limitation is that probes must avoid overlapping with primer‐targeted regions, leaving only a narrow sequence range suitable for SNP‐specific probe design. In addition, the fixed SNP sites often do not happen to fall in the region where the probe can be designed. As shown in **Figure** **1**B, in LAMP's primer configuration strategy, only 1 or 2 separate 20 nt regions (the regions of loop primers) are available for probe design. Recently, some researchers found that the invasion of inner and loop primers is the rate‐limiting step in the entire LAMP reaction.^[^ ^13^ ^]^ Basing this, we attempted to developed a smart LAMP method by modifying the BIP primer to allow great flexibility in SNP probe design, with the range of regions that can be designed being expanded from ≈20 to ≈60 nt. As described above and in Figure 1C, the primer FIP invades the dsDNA template with the assistance of primer LF and initiated the reaction. Next, the ssDNA product extended from FIP was displaced by the elongation of F3, providing a template for LF, B2, and B3. Two kinds of single‐stranded DNA (ssDNA) formed by the extension of LF and B2 will be displaced as B2 and B3 extended. At ≈60 °C, this short DNA fragments (≈60 bp) undergo dsDNA breathing, reaching a dynamic equilibrium between double‐stranded and single‐stranded structures. Hence, the ssDNA form by LF extension can be hybridized with FIP and a short double‐stranded DNA (dsDNA) is formed. Subsequently, LF binds during dsDNA breathing and generates new ssDNA amplicons. These ssDNA become new templates and the reaction cycles continue (Cycle I). On the other side, the ssDNA formed by the extension of B2 forms a single‐loop DNA double‐stranded structure, which FIP and B2 can easily hybridize to and produce new ssDNA. With the help of LF, the same single‐loop DNA double‐stranded structure was formed, thereby initiating another cycling reaction (Cycle II).

**Figure 2:**
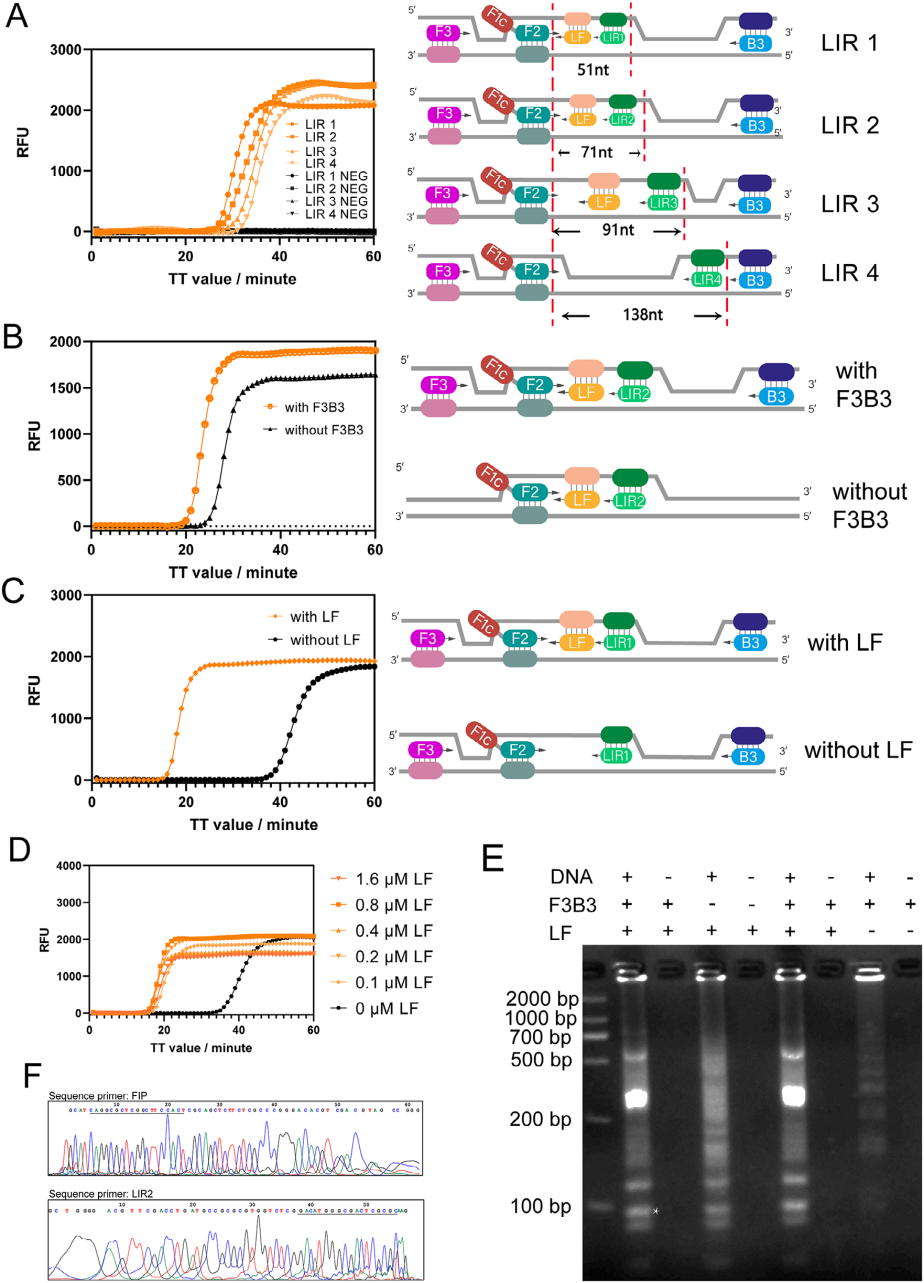
The validation of the principle of ssLAMP method. A) Evaluation of the effect of amplification product length in the ssLAMP system. B) Evaluation of the effect of F3B3 primers in the ssLAMP system. C) Comparison of ssLAMP amplification with LF primer and without LF primer. D) Optimization of the concentration of LF primer. E) Agarose gel electrophoresis analysis of ssLAMP method driven by different primer sets. The asterisk (*) marks the specific band that was excised from the gel and subjected to sequencing analysis. F) Sequence analysis of expected bands for the ssLAMP method. Bands were sequenced using the FIP and LIR2 primer.

We then evaluated the ability of ssLAMP to amplify DNA sequences of different lengths. It was found that DNA sequences ranging from 60 to at least 140 bp in length can be efficiently amplified within 40 min (**Figure** **2**A). The necessity of the outer primers (F3 and B3) was also evaluated. As shown in Figure 2B, the absence of outer primers in the reaction system reduced the efficiency of nucleic acid amplification. To develop a more robust ssLAMP reaction system, we explored the effects of accelerating primer LF. According to the ssLAMP principle, we speculated that it might be accelerated by the invasion of LF in the dsDNA. When adding the LF primer, threshold time (TT) values of ssLAMP were markedly reduced (Figure 2C). This proved that an extra LF primer was effective at accelerating ssLAMP, consistent with our prediction that LF plays an important role in accelerating the rate of initiation of ssLAMP. To evaluate the stability of ssLAMP amplification by adding LF primer, we tested the effect of different concentrations of LF primer on the reaction. The result showed that with a concentration of LF primer up to 1.6 µm, there are no non‐specific results, indicating the stability of ssLAMP amplification with LF primers (Figure 2D).

We further analyzed the reaction products of ssLAMP using agarose gel electrophoresis. Typical ladder‐like product patterns of isothermal amplification can be observed (Figure 2E). DNA sequence analysis confirmed that the result matched the expected product sequence (Figure 2F).

### 2.2 Comparison of ssLAMP Method and LAMP Based Methods

**Figure 3:**
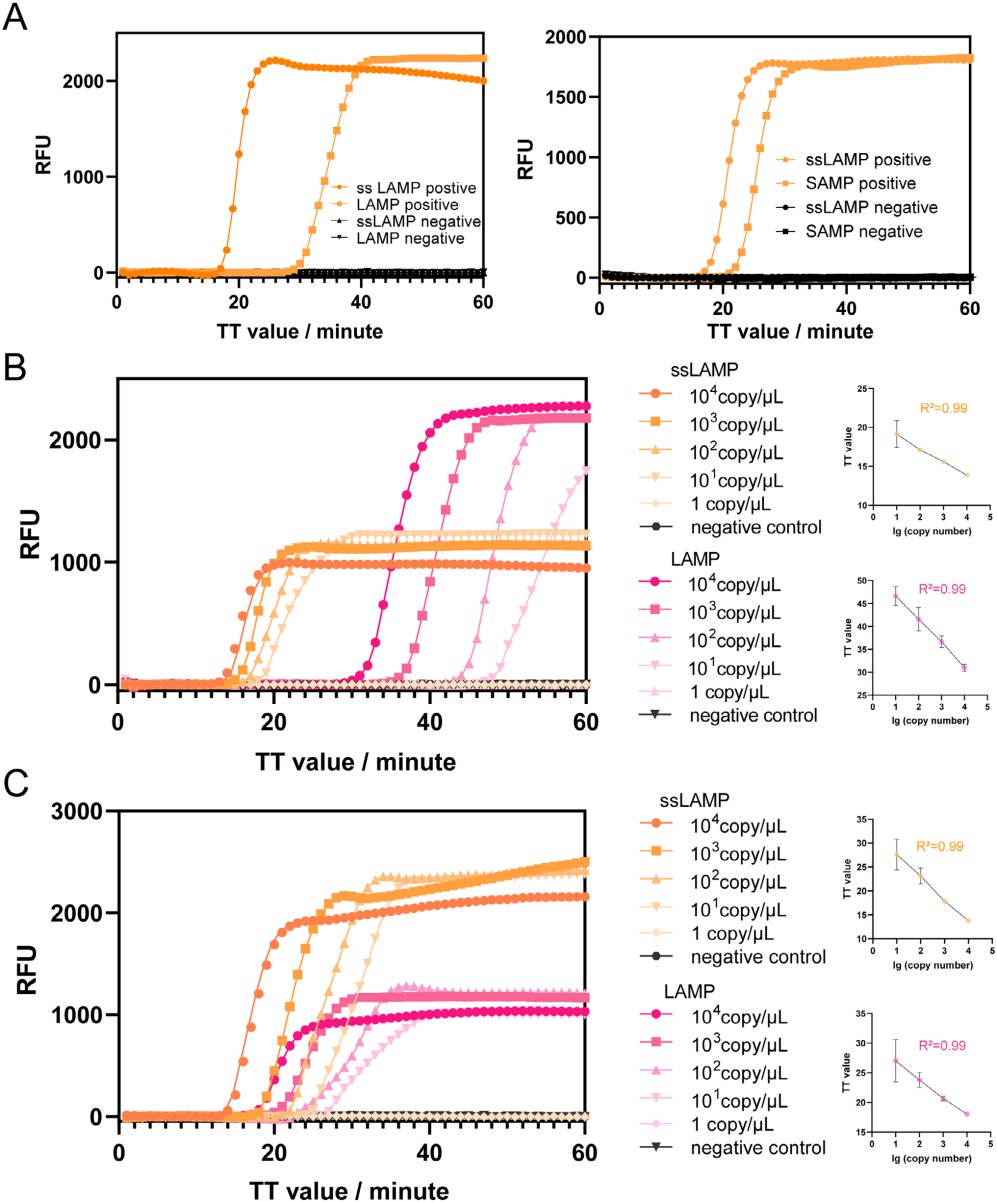
Effects of accelerating primers and optimization of reaction conditons on ssLAMP. A) Comparison with ssLAMP, LAMP, and SAMP. B) Sensitivity test of ssLAMP and LAMP with high GC contant (*gE* gene) plasmid template and linear regression between the indicated dilutions of plasmid and TT value. C) Sensitivity test of ssLAMP and LAMP with low GC contant (*invA* gene) plasmid template and linear regression between the indicated dilutions of plasmid and TT value.

To evaluate the efficiency of ssLAMP, we compared its performance with the LAMP method using the same plasmid template. The operable temperature range of ssLAMP was evaluated, demonstrating robust performance across a temperature spectrum of 58 to 65 °C (Figure S1, Supporting Information). We were surprised to find that the amplification signal appeared earlier in ssLAMP than in LAMP (**Figure** **3**A). Meanwhile, ssLAMP showed faster reaction efficiency than SAMP,^[^ ^11^ ^]^ another version of LAMP method (Figure 3A; Figure S2, Supporting Information).

Then, the sensitivity of ssLAMP was evaluated using tenfold dilutions of dsDNA. Meanwhile, a LAMP method targeting the same region of *gE* gene was designed for comparison. The results in Figure 3B showed that the detection limit of ssLAMP was as low as 100 copies/µL, which was comparable to LAMP. Notably, for the same concentration of template, the signal of the ssLAMP response appeared earlier. The ssLAMP method can detect DNA templates with the concentration of 10 copies/µL in ≈30 min, while LAMP needed ≈60 min to generate the amplification signal. Further, we test ssLAMP method using *invA* gene as a target gene, which has a relatively low GC content (46%). The result shows that ssLAMP had the same detection limit (10 copies/µL) as the LAMP method (Figure 3C), while the signal response also appeared earlier, consistent with that of the *gE* gene.

### 2.3 Applicability of ssLAMP Method Targeting SARS‐CoV‐2 *S* Gene

We designed corresponding probes according to these mutation sites (BA.1 probe for ins214EPE; BA.1/3 probe for N211del, L212I; BA.1/2/3 probe for Q493R; BA.4 probe for N658S; BA.4/5 probe for F486 V). Then, we tried to design LAMP primers that are suitable for these probes, but we found no primers could be produced if these probes were located on the loop primer region by using the online software. So, we tried to design ssLAMP primers according to its principles. The detailed information of primer sets and probes are shown in Table S1 (Supporting Information).

For each mutation sites, probes modified with a ribonucleotide were designed to specifically target to the alleles of the wild type or the mutant. Based on the characteristic that Bst DNA polymerase does not have 3′ to 5′ exonuclease activity, we introduced Rnase H2 in the ssLAMP reaction to cleave the probe. Only when the modified ribonucleotides in the probe perfectly match the template will the probe be cleaved, producing a fluorescence signal accompanied by the separation of the quencher dye and the fluorescent dye. The probe is modified with a C3 Spacer group at the 3′ end. The C3 Spacer modification at the 3′ terminus effectively blocks polymerase‐mediated extension of the probe, preventing nonspecific amplification during template‐directed DNA synthesis.

**Figure 4:**
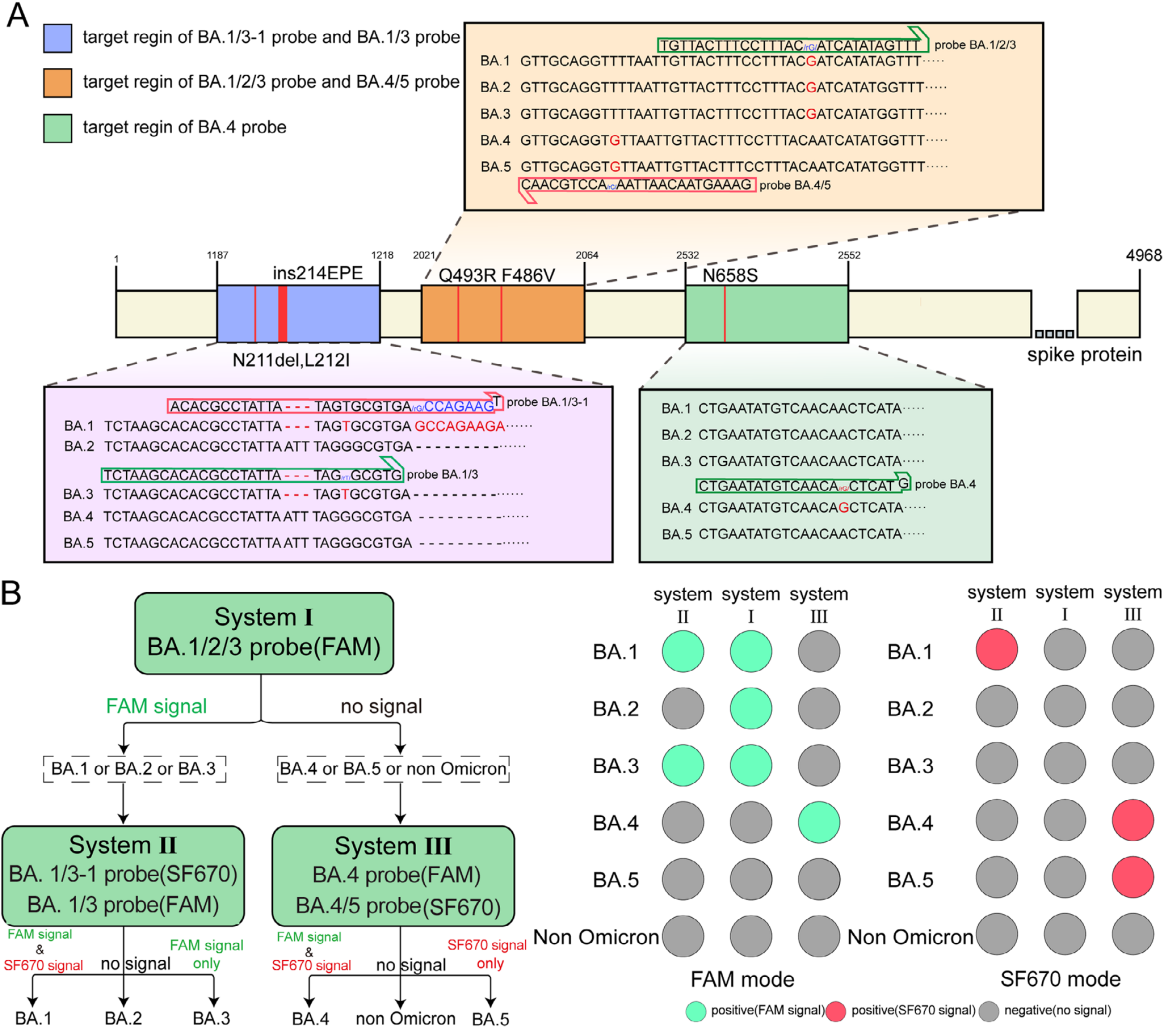
Working principle of ssLAMP‐based rapid testing of the SARS‐CoV‐2 Omicron variant. A) Schematic of SARS‐CoV‐2 S gene with annotation of the mutations or region targeting different VOC (highlighted in red colors). The specific mutations or region recognized by the different probes are also shown for each detection system. B) Genotyping strategy for SARS‐CoV‐2 Omicron variants with 3 individual reaction systems.

To validate this approach, we develop individual reaction systems to evaluate the ability of probes detecting these targets separately. All primer‐probe sets correctly discriminated their target mutation sites (Figure S3, Supporting Information). The limit of detection (LOD) of all the mutations are as low as 100 opies/µL (Figure S4, Supporting Information). The good linear regression for the dilution series (R^2^ > 0.97) (CV < 5%) revealed the reliability of the quantification method. Then, primers and probes were assembled and divided into 3 different multiplex reaction system (**Figure** **4**A). Reaction system I contained a FAM‐labeled BA.1/2/3 probe for the detection of BA.1, BA.2, and BA.3. Reaction system II contained a SF670‐labeled BA.1 probe and a FAM‐labeled BA.1/3 probe for the detection of BA.1 and BA.3. Reaction system III contained a FAM‐labeled BA.4 probe and a SF670‐labeled BA.4/5 probe for the detection of BA.4 and BA.5. The principle of multiplex ssLAMP for SARS‐CoV‐2 Omicron variants genotyping was proposed according to the mutation site among different variants. The 493R site was used to distinguish BA.1, BA.2, and BA.3 from BA.4 and BA.5. When the reaction system I produced FAM signal, the sample was judged as BA.1 or BA.2, or BA.3. On this basis, the typing results were obtained according to the signal of system II (with both FAM and SF670 signal for BA.1; only FAM signal for BA.3; without signal for BA.2). If no signal produced from reaction system I, the detection result is also obtained by observing the corresponding signal from reaction system III (with both FAM and SF670 for BA.4, only FAM signal for BA.5).

**Figure 5:**
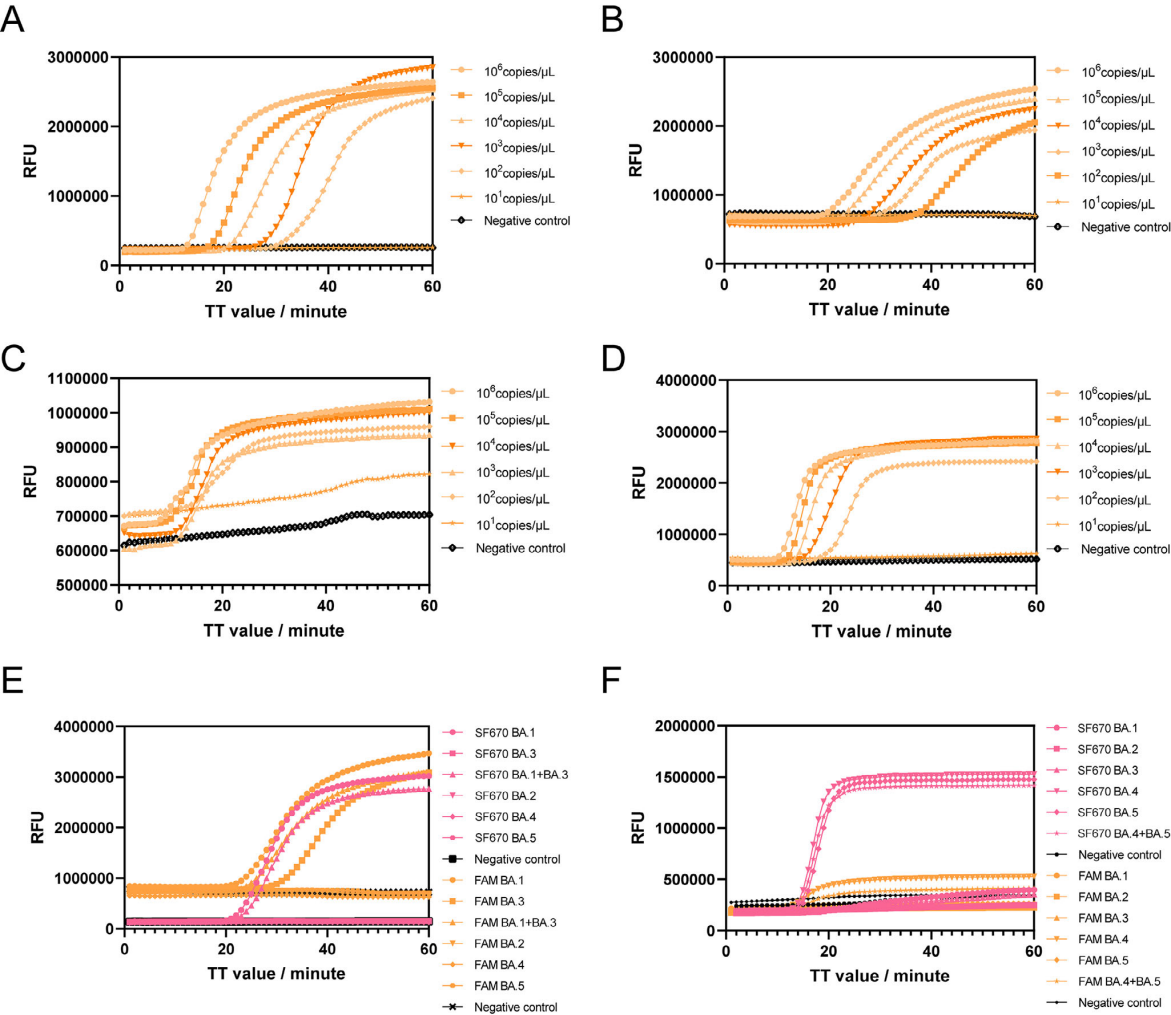
Sensitivity and specificity tests of ssLAMP‐based rapid testing of the SARS‐CoV‐2 Omicron variants. A) Sensitivity test of the systemII multiplex ssLAMP assay with BA.1 plasmid. B) Sensitivity test of the systemII multiplex ssLAMP assay with BA.3 plasmid. C) Sensitivity test of the systemIII multiplex ssLAMP assay with BA.4 plasmid. D) Sensitivity test of the systemIII multiplex ssLAMP assay with BA.5 plasmid. E) Specificity test of the systemII multiplex ssLAMP assay with different plasmid. F) Specificity test of the systemIII multiplex ssLAMP assay with different plasmid.

We constructed plasmids containing different mutation sites as reaction templates to evaluate the specificity and sensitivity of the ssLAMP method. The multiplex ssLAMP assay showed not only good specificity to individual or mixture target, with the LOD of 100 copies/µL (**Figure** **5**), but also good linear regression for the dilution series (Figure S5, Supporting Information). The results demonstrated that the multiplex ssLAMP assay exhibits excellent performance in detecting various Omicron variants.

A comparative analysis was performed to evaluate the specificity of ssLAMP and conventional LAMP method for detecting the ins214EPE and L212I mutations (reaction system II of ssLAMP). As shown in Figure S6 (Supporting Information), ssLAMP accurately distinguished different type of plasmid templates, whereas conventional LAMP failed to do so and exhibited non‐specific fluorescence signal curves.

### 2.4 ssLAMP Based POCT Using a Portable Device and Smart Phone

**Figure 6:**
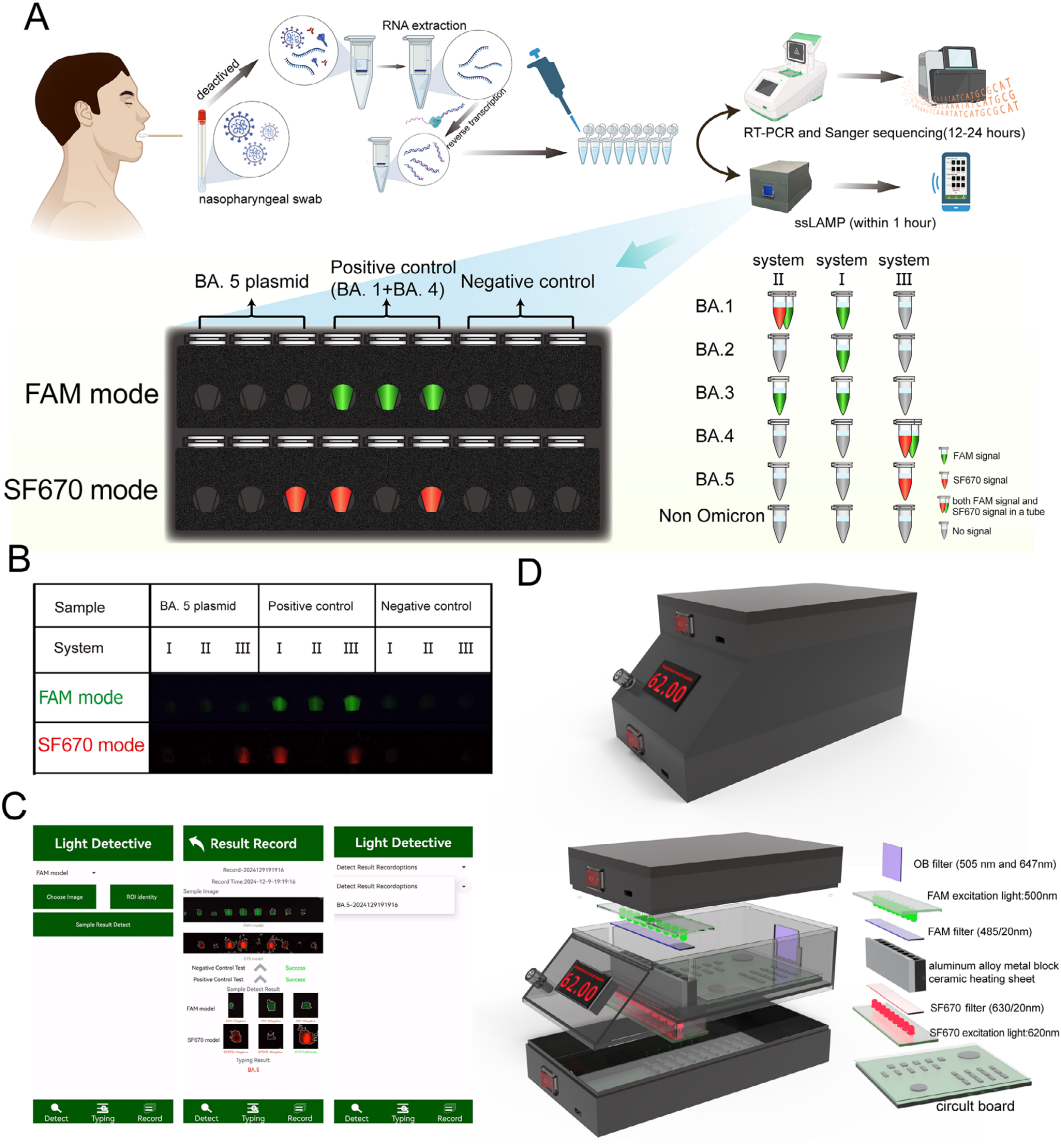
Point‐of‐care testing of SARS‐CoV‐2 variants using ssLAMP method with a portable device and a smart phone. A) Workflow of SARS‐CoV‐2 Omicron variant detection in clinical swabs when using the ssLAMP assay combine with the portable device and smart phone. B) Plasmid sample tested by ssLAMP and the protable device. C) Interface of the ssLAMP smart analyzing APP. D) 3D rendering of the protable detection device. Parts of the image were designed with BioRender.com, with permission.

To realize the POCT application of this method, a dual‐fluorescence detecting device (**Figure** **6**D; Figure S7, Supporting Information) was designed to facilitate visual interpretation of the results. The portable device could provide stable temperature control for ssLAMP reactions (Figure S8, Supporting Information). Fluorescence signals were captured using a smartphone by observing through the device's filter. For in situ detection, systems I‐III were placed in 3 separate reaction tubes. The negative control group used ddH₂O as the template, while the positive control group used a plasmid mixture of BA.1, BA.4, and BA.5. When the negative control group did not produce signals, system I‐III produced FAM signals, and the system II and system III produced SF670 signals in the positive control group, it indicated that the reaction system was running correctly. As shown in Figure S9 (Supporting Information), BA.1‐BA.5 plasmid could be correctly recognized by reaction systems I‐III. The detection result can be judged by the signal pattern generated by the sample to be tested in the FAM channel and SF670 channel. Moreover, the LOD of ssLAMP when utilizing the portable device (Figure S9, Supporting Information) was found to be identical to that achieved through the use of the Applied Biosystems 7500 real‐time PCR System (Figure 5).

A mobile application was developed to analyze the fluorescent signals collected from the samples. The application was written in Java and developed in the Android Studio Integrated Development Environment with a OnePlus 8 smartphone as the test device. The custom APP assists in determining test results by analyzing the RGB color channel attributes associated with FAM and SF670 signals, and providing conclusions on the typing results of positive samples. The application contains three main functions: Detect, Typing, and Record. The APP analyses the FAM and SF670 fluorescence signals through a series of image processing steps, the main processes of which are as follows: 1) Edge detection: The Canny algorithm is utilized to extract edges in regions of the image exhibiting significant changes in gradient, thereby accurately locating the target boundaries. 2) Color segmentation: The image is converted into the HSV color space, and the color features of the corresponding fluorescence signals (FAM for green, SF670 for red) are extracted by means of threshold filtering. 3) Morphological operations: The reduction of noise and enhancement of the target region's boundary is achieved through morphological operations such as expansion and erosion, thereby enhancing the segmentation's robustness. 4) Contour Detection: The contour boundaries of the target region are detected following morphological processing to identify regions of interest (ROIs). 5) Region Segmentation: Each target region is individually segmented based on the detected contours to facilitate subsequent independent analysis. Following the completion of region segmentation, the application calculates the RGB average pixel values (R_avg, G_avg, B_avg) for each ROI, excluding purely black pixels to avoid calculation errors. The detection value is calculated by Equations (1) and (2), with the FAM signal calculated by Equation (1) and the SF670 signal by Equation (2). The detection threshold is determined as the average signal value of the control group plus three times the standard deviation. If the detection value of the sample exceeds this threshold, it is determined as positive; otherwise, it is determined as negative. During the typing process, when FAM and SF670 signals are detected, the application first validates the detection results of the control samples. If the negative and positive control groups run correctly, the typing results are output according to the predefined typing strategy (Figure 4B); otherwise, the typing fails. All analysis results are saved and can be displayed for user review.

(1) $$\frac{G_{avg}}{R_{avg}+G_{avg}+B_{avg}}$$

(2) $$\frac{R_{avg}}{R_{avg}+G_{avg}+B_{avg}}\;$$

* *R_avg_* represents the average value of all pixels under the R channel. *G_avg_* represents the average value of all pixels under the G channel. *B_avg_* represents the average value of all pixels under the B channel

### 2.5 Evaluation of the ssLAMP Assay in the Rapid Differentiation of SARS‐CoV‐2 Omicron Variants

**Figure 7:**
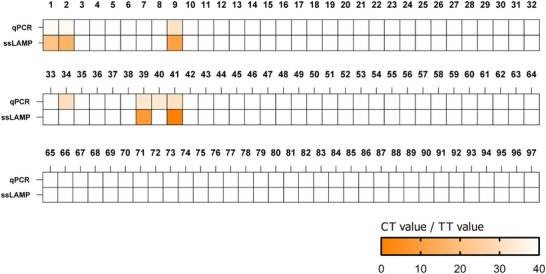
Comparison between qPCR method and ssLAMP‐based rapid testing of clinical nasopharyngeal swab sample.

To evaluate the robustness and accuracy of ssLAMP with real samples, 97 NP swab samples collected from suspected patients with respiratory symptoms at the First Hospital of Jilin University were tested. Among these, ssLAMP detected 5 positive samples of BA.5 sublineage (**Figure** **7**; Figures S10–S19, Supporting Information). The RT‐qPCR assay recommended by the Chinese Center for Disease Control and Prevention was used as a control assay. Compared with RT‐PCR, ssLAMP showed 100% concordance in negative results. All the positive samples detected by ssLAMP were also confirmed as positive by RT‐qPCR and Sanger sequencing (Figure S20, Supporting Information). However, two samples identified as positive by RT‐qPCR were not detected by ssLAMP. It is speculated that this discrepancy may have resulted from a low SARS‐CoV‐2 load in the NP swab or by the presence non‐Omicron lineages.

## 3 Discussion

The high mutation rate of SARS‐CoV‐2 has led to frequent alterations in transmissibility and immune evasion properties, posing significant challenges for the development of vaccines and drugs. Hence, accurate identification and epidemiologic surveillance of different SARS‐CoV‐2 variants are essential for effective prevention and control of SARS‐CoV‐2. Current detection methods, such as PCR and traditional LAMP, often fail to meet the demands for rapid, on‐site, and multiplex detection of SARS‐CoV‐2 variants. Recent advancements in amplification‐free nucleic acid detection have shown promise, such as deep‐learning‐assisted holographic microscopy platforms for single‐particle counting, which achieve 72 CFU mL^−1^ sensitivity without target amplification,^[^ ^14^ ^]^ and Argonaute‐mediated digital sensors enabling multiplex pathogen detection at 6 CFU mL^−1^ through AI‐driven magnetic bead decoding.^[^ ^15^ ^]^ These studies innovatively integrate CRISPR‐Cas, argonaute‐based nucleic acid signal amplification systems with artificial intelligence and portable detection devices, enabling amplification‐free nucleic acid testing for multiplex pathogen targets. These approaches have emerged as a next‐generation strategy to accelerate the translation of accurate and convenient pathogen detection technologies from laboratories to POCT. However, for highly mutable pathogens such as SARS‐CoV‐2, achieving precise genotyping not only requires the detection of target gene fragments but also necessitates the acquisition of SNP information at relevant mutation sites. In this study, we developed a novel nucleic acid isothermal amplification method, termed smart single‐loop‐mediated isothermal amplification (ssLAMP), which surprisingly showed higher reaction efficiency than traditional LAMP method through revamped primer sets. More importantly, the smartly primer sets design strategy in ssLAMP provides greater flexibility for placing SNP detection probes in free regions between primers. Combining with a portable detection device and smartphone APP, the ssLAMP enables differentiation of SARS‐CoV‐2 Omicron variants including BA.1, BA.2, BA.3, BA.4, and BA.5 without relying on DNA sequencing.

In contrast to the PCR method which can rely on higher reaction temperatures to facilitate the denaturation of DNA double strands, LAMP is typically conducted at temperatures ≈60 °C. Double strands DNA cannot be denatured completely under this temperature, while the DNA breathing phenomenon^[^ ^16^ ^]^ occurs, leading to a state of equilibrium between double‐stranded DNA and single‐stranded DNA configuration in some regions. According to kinetics of elementary steps in LAMP, a variety of accessory primers are critical to enhance primer invasion of double‐stranded targets, presumably by facilitating strand separation.^[^ ^13^, ^17^ ^]^ Typically, 6 to 8 regions of ≈20 nt in length (usually are F1c, F2, F3, B1c, B2, B3; LF, and LB if available) in a region of ≈250 bp of the target sequence are required to meet the requirements in traditional LAMP primer design, including adequate GC content (40–60%), relatively consistent melting temperatures, and especially avoidance of primer dimers and hairpin structures, etc. However, the current requirements are stringent and cumbersome, significantly increasing the difficulty of LAMP primer design. In some cases, it is not even possible to assemble a compliant LAMP primer set when facing special sequences, such as those with an excessively high or low GC content. In addition, this compact primer arrangement in a limited area leaves a very limited space for SNP probe insertion. These challenges highlight the need for a more flexible and efficient isothermal amplification method, which led us to develop ssLAMP.

To address these challenges, we developed the ssLAMP by replacing the inner primer (FIP or BIP) with a linear inner primer (Figure 1A). The design strategy and combination pattern of primer sets by removing the inner primer on one side to reduce the primer target region. This novel primer design offers two key advantages. First, by discarding an inner primer that can form a stem‐loop structure in the reaction, the primer complexity is reduced thereby reducing the potential for primer dimerization and non‐specific binding of the primer to the template. The intricate primer configuration of conventional LAMP frequently results in nonspecific amplification during duplex detection with two primer sets in a single‐tube reaction. The ssLAMP was designed successfully implementing the integration of the two primer sets BA.4 and BA.5 in a single‐tube reaction without the generation of a nonspecific amplification signal. Besides, we found that addition of a accelerated primer (LF or LB) can facilitate its amplification ability to the same as normal LAMP, but faster than SMAP, a smart LAMP previously reported.^[^ ^11^ ^]^ The incorporation of accelerated primers in the case of a reduced number of primers ensured the efficacy of the ssLAMP reaction. Second, this approach can also provide more space for the insertion of SNP probe, making the detection of SNP sites in different regions easier. Sometimes, we found primers of the normal LAMP were overlapped with the SNP probe, which would make the SNP probe combine with other primers then would general non‐ specific fluorescent signal during the LAMP reaction. The ssLAMP provided a suitable region for us to insert two probes (probe BA.1 and probe BA.1/3) into a single primer set simultaneously, enabling dual detection of the BA.1 and BA.3 SNP sites. Furthermore, ssLAMP exhibits greater flexibility in primer design than traditional LAMP, as fewer areas are necessary to meet the requisite specifications. The ssLAMP method we developed was successfully applied to templates with relatively low GC content (*invA* gene, GC content 46%) and high GC content (*gE* gene, GC content 74%). This enables ssLAMP to demonstrate great compatibility and primer design flexibility for different target genes.

In this study, end‐point determination of SARS‐CoV‐2 Omicron variants was realized by using 3D‐printed dual‐fluorescence visualization device with isothermal control function. The ssLAMP method for SARS‐CoV‐2 Omicron variants detection exhibited satisfactory sensitivity (100 copies/µL) and high specificity for the single‐base mutations detection of BA.1, BA.2, BA.3, BA.4, and BA.5 variants within a 90‐min reaction time. In clinical sample testing, the results of the ssLAMP were consistent with the results of Sanger sequencing combined with PCR, confirming its reliability. To the best of our knowledge, this is the first reported multiplex POCT methods for SARS‐CoV‐2 Omicron variants. Previous reported detection methods, predominantly PCR‐based, are time‐consuming, complicated and require specialized technicians.^[^ ^18^ ^]^ Although some POCT technologies were reported, most of them fail to achieve multiplex and high‐throughput detection.^[^ ^12^ ^]^ In contrast, the multiplex ssLAMP method described here is automated via matched software and demonstrates potential for high‐throughput applications This cost‐effective POCT approach for SARS‐CoV‐2 Omicron variants is sensitive and specific, making it suitable for on‐site diagnostics or resource‐limited settings.

In conclusion, the ssLAMP method offers significant advantages due to its flexible primer design. In addition to its compatibility with SNP‐based RNase H2‐activated probes, ssLAMP can be adapted for use with other probe‐based detection systems. This versatility positions ssLAMP as a promising isothermal amplification method for POCT applications. However, ssLAMP remains limited by its lower sensitivity compared to qPCR methods. Future research will integrate CRISPR‐Cas or Argonaute‐mediated multiplex recognition, and digital microfluidic platforms to develop rapid, ultra‐sensitive field detection systems. We believe that this approach can become a promising isothermal nucleic acid amplification method for POCT applications.

## 4 Experimental Section

### Regents

Lyo‐ready Bst DNA Polymerase (30 µL, 40 U/µL), 10× Bst Reaction Buffer (1 mL), 200 mm MgCl_2_ (1 mL), 5 m Betaine (1 mL) were purchased from Thermo Fisher Scientific (USA). A 10 mm deoxynucleotide (dNTP) was obtained from Vazyme Biotech (China). Eva Green dye was procured from Biotium Inc. (USA).

### DNA and RNA Extraction

Pseudorabies virus (PRV) GD‐WH and *Salmonella* Typhimurium (ATCC14028) were obtained from laboratory stocks. Viral DNA was extracted using a HiPure Viral RNA/DNA Kit (Magen, China) and viral RNA was purified using a GeneJET RNA Purification Kit (Thermo Fisher Scientific, USA) according to the instructions of the manufacturer. Bacterial DNA was extracted using HiPure Bacterial DNA Kit (Magen, China) according to the manufacturer's protocol. The purified DNA and RNA were dissolved in nuclease‐free water and stored at −80 °C.

### Standard Template Preparation

The *gE* gene of PRV and the *invA* gene of *Salmonella* were amplified by PCR. The PCR products were purified using a Cycle Pure Kit (Omega, USA) following the manufacturer's instructions. The purified fragments were cloned into the pMDTM19‐T vector and transformed into DH5α competent cells (TaKaRa Biotechnology, China). Plasmid DNA was extracted using a Plasmid Mini Kit I (Omega) and used as the template for subsequent reactions. The S gene plasmids of SARS‐CoV‐2 Omicron variants (BA.1, BA.2, BA.3, BA.4, and BA.5) were synthesized and cloned into the pUC19 vector by Sangon Biotech (China).

### Primer Design

Primers for the ssLAMP method targeting the SARS‐CoV‐2 *S* gene (NCBI Reference Sequence: NC_045512.2) were designed using PrimerExplorer V5 tool (http://primerexplorer.jp/lampv5e/index.html) and Primer Premier 5.0. LAMP primers for the PRV *gE* gene (GenBank: KT936468.1) and *Salmonella invA* gene (GenBank: 1 254 419) were designed using online PrimerExplorer V5. The designed primers are listed in Table S1 (Supporting Information). All primers were synthesized by Sangon Biotech.

### Development of ssLAMP and LAMP Reactions

The mixtures for ssLAMP and LAMP reactions were consistent with typical isothermal methods, and contained 1× Bst Reaction Buffer, 1× Eva Green dye, 1.4 mm dNTPs, and 6 U Bst WarmStart DNA polymerase. The concentration of ssLAMP primers was optimized as 1.6 µm FIP or BIP, 1.6 µm LIR or F2 or B2, 0.8 µm LF or LB, and 0.2 µm F3 or B3. The optimal concentration of LAMP primers was 1.6 µm FIP or BIP, 0.8 µm LF or LB, and 0.2 µm F3 or B3. Reactions were performed on a Roche Light Cycler 96 real‐time detection system (Roche, Switzerland) and an Applied Biosystems 7500 real‐time PCR System (Thermo Fisher Scientific, USA). Reactions consisted of 60 cycles at 63 °C for 1 min, with fluorescence signals measured at the end of each cycle.

### Sequence Identification of ssLAMP Products

After real‐time detection, ssLAMP products were analyzed by 3% agarose gel electrophoresis and stained with GoldView II Nuclear Staining Dye (Solarbio, China). The gel images were visualized and captured using the ChemiDoc XRS Imaging System (Bio‐Rad Laboratories, USA). After agarose gel electrophoresis analysis, the single band corresponding to the theoretical size was excised from the gel and purified using the Gel Extraction Kit (Omega, USA). The purified DNA was sequenced by Sangon Biotech.

### Sensitivity Analysis

Ten‐fold serial dilutions of plasmid DNA containing the PRV *gE* gene (10^6^, 10^5^, 10^4^, 10^3^, 10^2^, 10, and 1 copies) were prepared to evaluate the detection limit of the ssLAMP method in comparison with conventional LAMP. Negative controls were included for each reaction. Three independent replicates for each plasmid concentration gradient were conducted.

### Design of Probes Targeting to SNP Sites of SARA‐CoV‐2 Omicron Variants

The spike protein mutation sites used to identify SARA‐CoV‐2 Omicron variants (BA.1, BA.2, BA.3, BA.4, and BA.5) were obtained from GISAID databases (https:// gisaid.org).^[^ ^19^ ^]^ Probes targeting the SNP sites were designed using Primer Premier 5.0. Each SNP mutation site was placed in the middle of the probe and substituted by ribonucleotide. Detailed information about primers and probes is provided in Table S1 (Supporting Information).

Five independent ssLAMP reaction systems and 2 multiple reaction systems targeting Omicron BA.1, BA.2, BA.3, BA.4, and BA.5 were established. Reactions were run on the Applied Biosystems 7500 real‐time PCR System and were tested for analytical sensitivity and analytical specificity for plasmids of 5 SARS‐CoV‐2 Omicron sublineages. Three independent replicates for each plasmid concentration gradient were conducted in the sensitivity test.

### On‐Site Testing with Portable Testing Device

A portable testing device was designed for the on‐site testing of ssLAMP (Figure 6D; Figure S7, Supporting Information). The structure of the device was designed using SolidWorks 2019 software (Concord, MA, USA). The device dimensions were 180 mm (l) × 100 mm (w) × 94.2 mm (d). A high thermal conductivity aluminum alloy block served as the heating element, tightly connected to a positive temperature coefficient ceramic heating sheet through thermal conductivity silicone grease. To ensure precise excitation of the sample, the excitation lights of different wavelengths were placed in the upper and lower layers (the FAM excitation light: 500 nm; the SF670 excitation light: 620 nm). The LED array for excitation light was positioned directly above and below the high transmittance EP tube, and each EP tube corresponds to an LED lamp. The positioner with fixed parameters was used to fix the position of the excitation light LED and the EP tube, in order to eliminate the influence of the excitation light angle on the detection results. Additionally, a band‐pass filter, placed parallel to the LEDs, filtered unwanted emission wavelengths incapable of inducing fluorescence. A 9‐well module was incorporated in the device's middle layer for sample excitation and heating, with open apertures on the front of heating module facilitated the export of excited fluorescence.

### Human Clinical Specimen Collection and Ethics Statement

Clinical specimens were collected at the First Hospital of Jilin University. The Ethics Committee of the First Hospital of Jilin University ruled the ethics for all the clinical samples used in this study. The committee reviewed and approved the proposal for the collection and use of nasopharyngeal (NP) swab samples (22y050‐001). Swab samples collected and immediately inactivated by heating at 56 °C for 30–45 min before being stored at −80 °C for subsequent testing. RNA was extracted from 140 µL of input material using GeneJET RNA Purification Kit (Thermo Fisher Scientific, USA) following the manufacturer's protocol. Extracted RNA was eluted in 80 µL of nuclease‐free water and stored at −80 °C until use.

### The ssLAMP for SARS‐CoV‐2 Infected Clinical Samples Determination

RNA extracted from nasopharyngeal swab samples was reverse transcribed into cDNA using HiScript III All‐in‐one RT SuperMix kit (Vazyme, CHINA). The resulting cDNA was subsequently analyzed using the ssLAMP method for target detection. The ssLAMP reaction was performed in the custom‐manufactured portable device at a constant temperature of 62 °C for 60 min. The results were recorded by capturing images with a smartphone and subsequently analyzing them using software. For comparison, qPCR targeting SARS‐CoV‐2 ORF gene and N gene were also conducted. Nucleic acid sequence information of positive samples was verified by Sanger sequencing.

## Conflict of Interest

The authors declare no conflict of interest.

## Supporting information



Supporting Information

## Data Availability

The data that support the findings of this study are available from the corresponding author upon reasonable request.
