# Supplementary material for: A Smart Single‐Loop‐Mediated Isothermal Amplification Facilitates Flexible SNP Probe Design for On‐Site Rapid Differentiation of SARS‐CoV‐2 Omicron Variants
Source: Adv Sci (Weinh). 2025 Apr 1;12(26):2502708. doi: 10.1002/advs.202502708 (PMC12245020; doi:10.1002/advs.202502708)

Supporting Information for

**A Smart Single-Loop-Mediated Isothermal Amplification Facilitates Flexible SNP Probe Design for On-Site Rapid Differentiation of SARS-CoV-2 Omicron Variants**

*Qijie Lin, Hongchao Gou, Xiaoyun Qu, Kaiyuan Jia, Yuhui Deng, Dan Li, Qianyi Cai, Yucen Liang, Xiaozhen Xu, Yanbin Li, Jianhan Lin, Letian Li, Yuhang Jiang, Shouwen Du, Lingcong Deng, Bailing Yan, Ruidong Liu, Chang Li *, Jianmin Zhang *, Ming Liao **

**Table S1.** The primer sequences used in the ssLAMP method targeting different genes.

| **Group** | **Primer** | **Sequence (5'→3')** | **Length(nt)** | |
| --- | --- | --- | --- | --- |
| gE | F3 | ACGAGCCCCGCTTCCA | | 16 |
|  | B3 | AGATGCAGGGCTCGTACA | | 18 |
|  | LIR1 | AGACCACGCGCGGCATCAG | | 19 |
|  | LIR2 | GCGCGAGTCGCCCATGTC | | 18 |
|  | LIR3 | AGCGTGGCGGTAAAGTTCT | | 19 |
|  | LIR4 | CGTAGTACAGCAGGCACCG | | 19 |
|  | FIP | AGACCACGCGCGGCATCAGGCGCTCGGCTTCCACT | | 35 |
|  | LF | TGTCCCCGGGCGAGAAGA | | 18 |
| invA | F3 | GCGAAGCGTACTGGAAAGG | | 19 |
|  | B3 | TCAACAATGCGGGGATCTG | | 19 |
|  | LIR | AATAATGATGCCGGCAATAGCG | | 22 |
|  | FIP | ATGATGCCGGCAATAGCGTCACAAAGCCAGCTTTACGGTTCC | | 42 |
|  | LF | AAACTTCATCGCACCGTCAAA | | 21 |
| BA.4/5 | F3 | GCTTGATTCTAAGGTTAGTGG | | 21 |
|  | B3 | TCTGTATGGTTGGTGACC | | 18 |
|  | FIP | CGGCCTGATAGATTTCAGTTGAAATTTACCTGTATAGATTGTTTAGGAAG | | 50 |
|  | B2 | AACACCATAAGTGGGTCG | | 18 |
|  | LF | CTCTCAAAAGGTTTGAGATTAG | | 22 |
|  | Probe BA.4/5 | GAAAGTAACAA/iSF 670dT/TAA**/rC/**ACCTGCAAC/iSp C3/-BHQ3 | | 25 |
| BA.4 | F3 | AGGGTGTTAACTGCACAG | | 18 |
|  | B3 | GAGACTTAGTCTGAGTCTGAT | | 21 |
|  | BIP | CGTGCAGGCTGTTTAATAGGGCGCATATACCTGCACCAAT | | 40 |
|  | F2 | AAGTCCCTGTTGCTATTCATG | | 21 |
|  | LB | TATGTCAACAGCTCATATGAGTGTG | | 25 |
|  | Probe BA.4 | CTGAATATG/i6FAM dT/CAACA**/rG/**CTCATG/iSp C3/-BHQ1 | | 22 |
| BA.1/2/3 | F3 | AATCTATCAGGCCGGTAAC | | 19 |
|  | B3 | CATTGAAGTTGAAATTGACACAT | | 23 |
|  | LB | TTTGAACTTCTACATGCACCAGCA | | 24 |
|  | BIP | TTGGTCACCAACCATACAGAGTAAGACTTTTTAGGTCCACAAACA | | 45 |
|  | F2 | AACCTTGTAATGGTGTTGC | | 19 |
|  | Probe BA.1/2/3 | TGTTACTTTCC/i6FAM dT/TTAC**/rG/**A/iBHQ1dT/CATATGGTTT/iSp C3/ | | 29 |
| BA.1/3 | F3 | CCTTGAAGGAAAACAGGGTAA | | 21 |
|  | B3 | ACCAGGAGTCAAATAACTTCT | | 21 |
|  | LB | GAACCATTGGTAGATTTGCCAATAG | | 25 |
|  | BIP | AAGATCTCCCTCAGGGTTTTTCGAAAGTTTGAAACCTAGTGATGTT | | 46 |
|  | F2 | CAAAAATCTTAGGGAATTTGTGT | | 23 |
|  | Probe BA.1 | ACACGCCTATTATAG/iSF 670dT/GCGTGA**/rG/**CCAGAT/isp C3/-BHQ3 | | 29 |
|  | Probe BA.1/3 | TCTAAGCACACGCC/i6FAM dT/ATTATAG**/rU/**GCGTG/iSp C3/-BHQ1 | | 28 |

**Table S2.** The primer sequences of Taqman-qPCR method for SARS-CoV-2.

| **Target Region** | **Primer** | **Sequence (5'→3')** | **Length(nt)** |
| --- | --- | --- | --- |
| ORF1ab | F | CCCTGTGGGTTTTACACTTAA | 21 |
|  | R | ACGATTGTGCATCAGCTGA | 19 |
|  | Probe | 5'-FAM-CCGTCTGCGGTATGTGGAAAGGTTATGG-BHQ1-3' | 28 |
| N gene | F | GGGGAACTTCTCCTGCTAGAAT | 22 |
|  | R | CAGACATTTTGCTCTCAAGCTG | 22 |
|  | Probe | 5'-FAM-TTGCTGCTGCTTGACAGATT-TAMRA-3' | 20 |


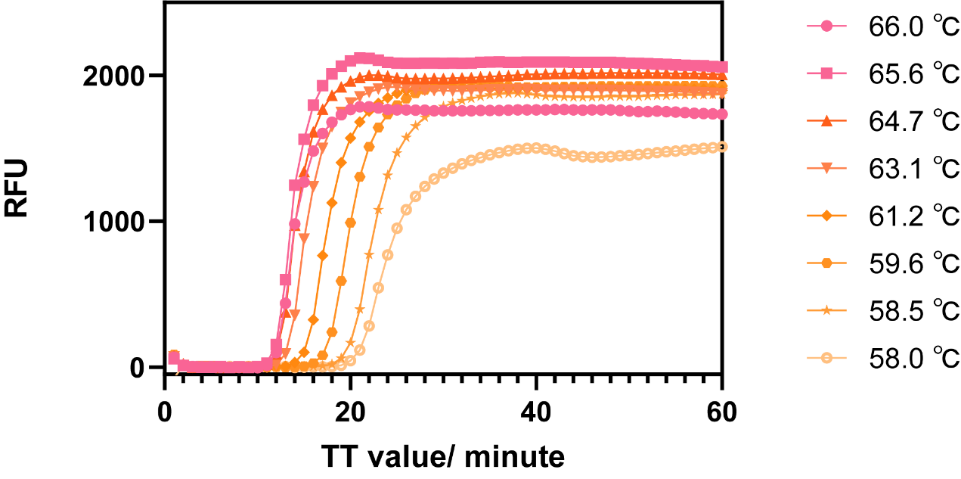


**Figure S1. Feasibility test of ssLAMP Amplification under different temperature conditions.**


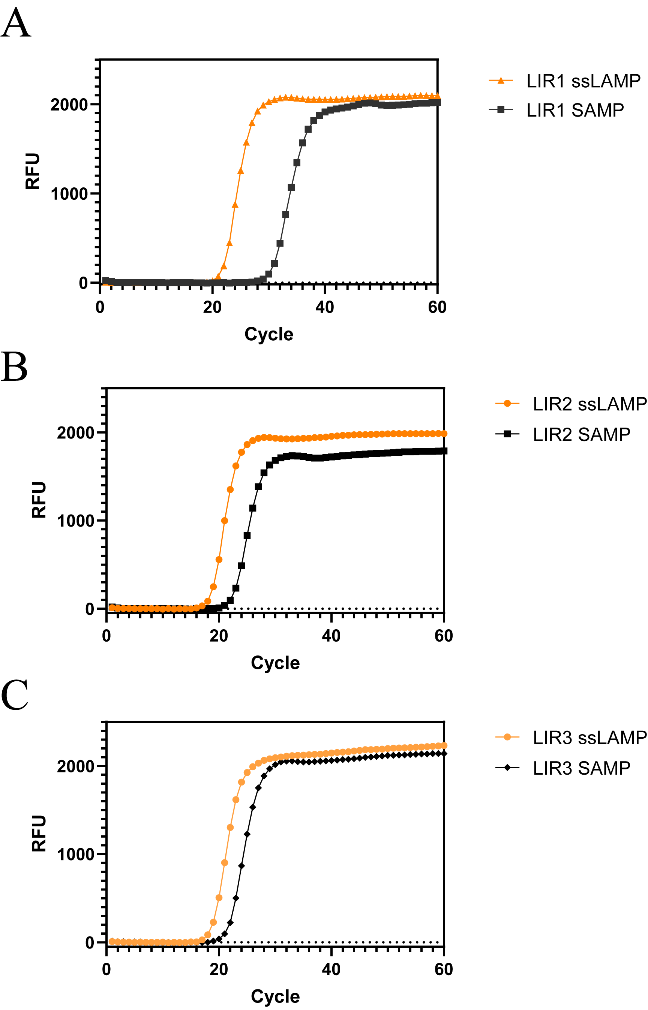


**Figure S2. Comparison of ssLAMP and SAMP Methods Using inner primers at different positions.** A, B and C are the comparison of ssLAMP and SAMP with inner primers LIR1, LIR2 and LIR3 resepctively.


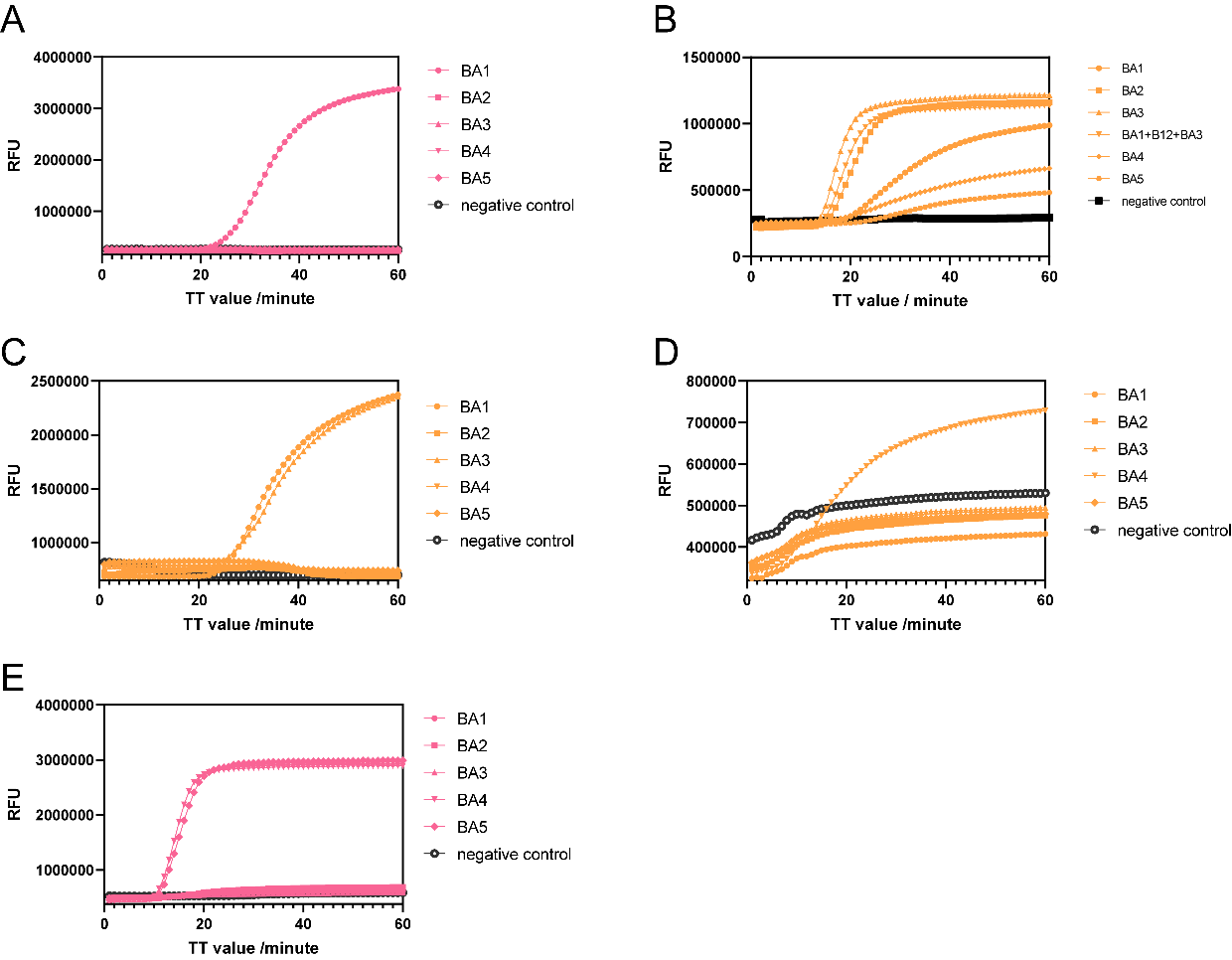


**Figure S3. The specific test of the ssLAMP using plasmid templates with different SNP sites.** A, B, C, D, and E are the specific tests of ssLAMP with BA.1 probe, BA.1/2/3 probe, BA.1/3 probe, BA.4 probe, BA.4/5 probe and related primers, respectively.


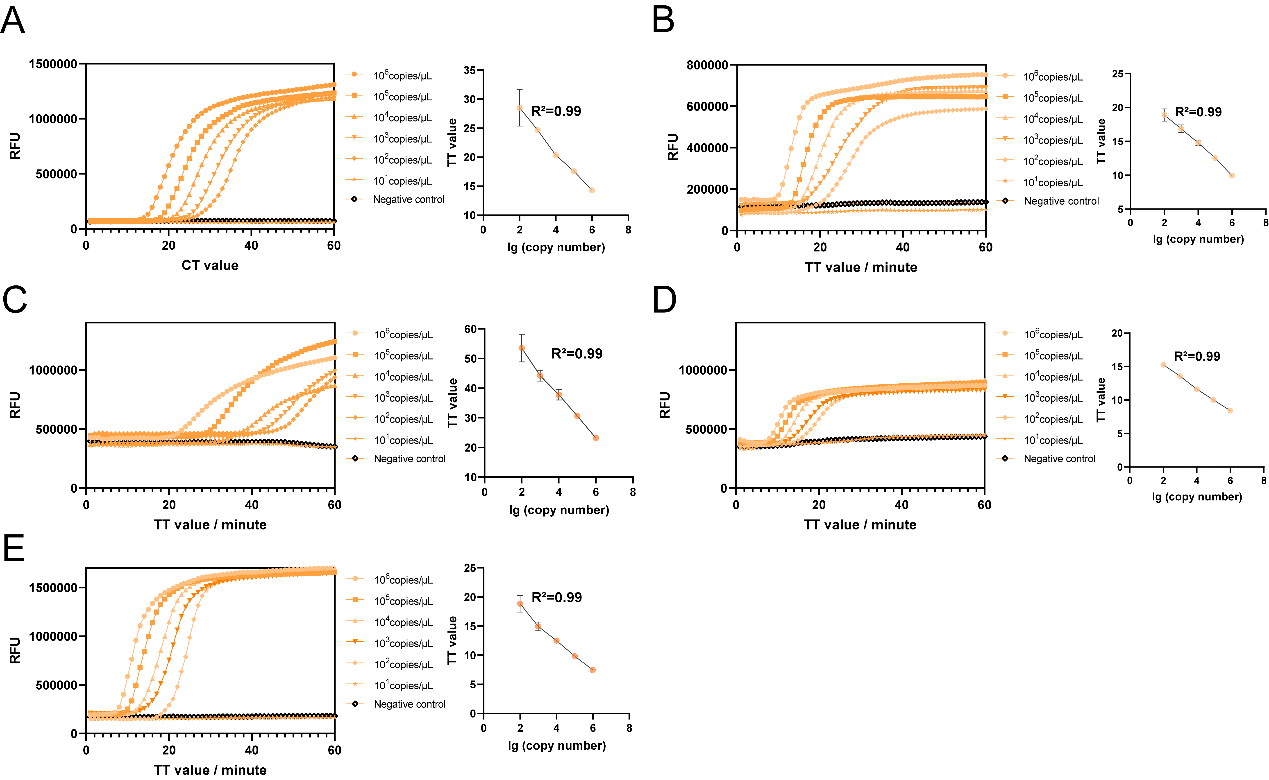


**Figure S4. The sensitivity test of ssLAMP using different plasmid templates with gradient concentration, and the linear regression between the indicated dilutions of plasmid and TT value.** A, B, C, D, and E are the ssLAMP with BA.1 probe, BA.1/2/3 probe, BA.1/3 probe, BA.4 probe, BA.4/5 probe and related primers, respectively.


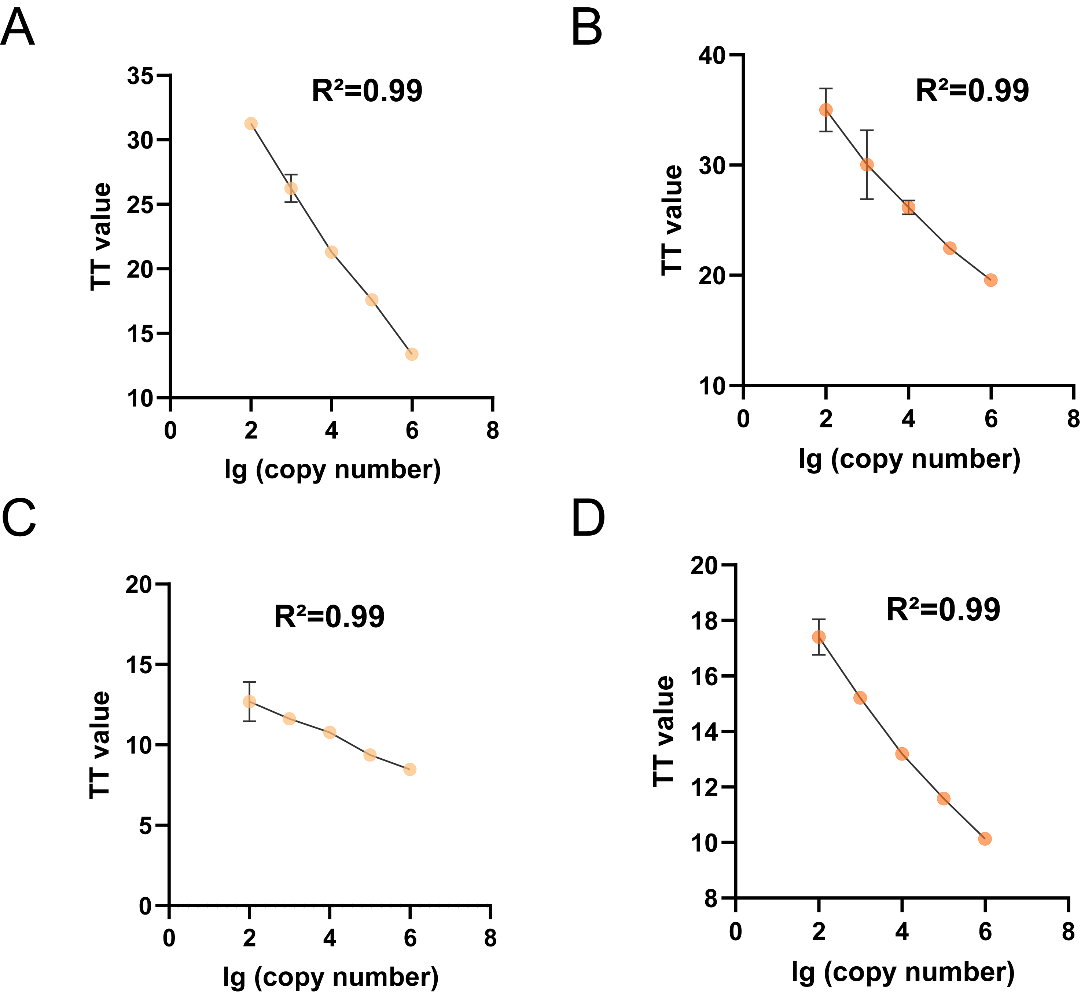


**Figure S5. The linear regression between the indicated dilutions of plasmid and TT value of different ssLAMP reaction system in multiplex** **SARS-CoV-2 Omicron variants detection.** A) system Ⅱ multiplex ssLAMP assay with BA.1 plasmid. B) system Ⅱ multiplex ssLAMP assay with BA.3 plasmid. C) system Ⅲ multiplex ssLAMP assay with BA.4 plasmid. D) system Ⅲ multiplex ssLAMP assay with BA.5 plasmid.


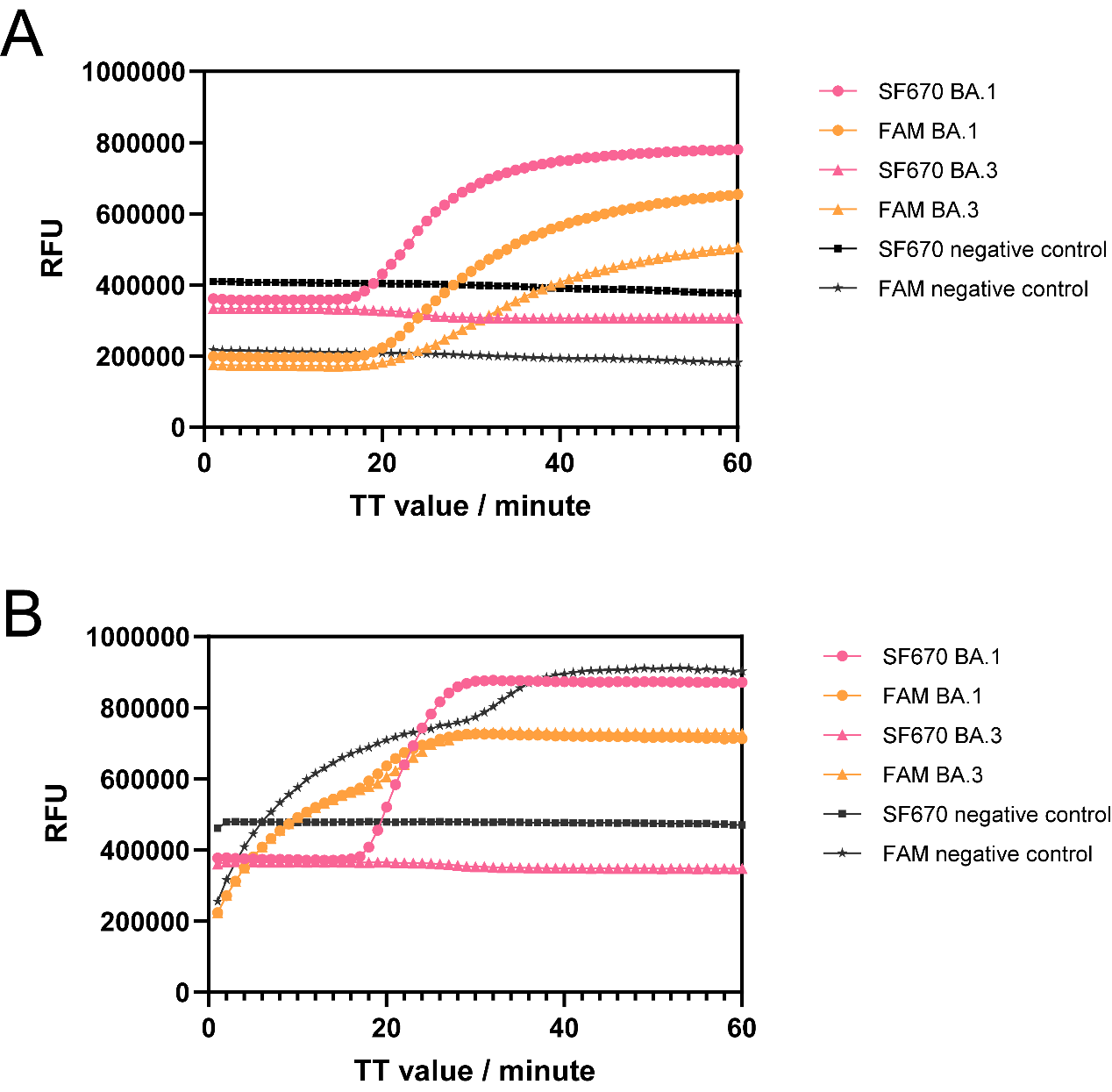


**Figure S6. The specificity tests of ssLAMP and conventional LAMP method for detecting the ins214EPE and L212I mutations.** A) system Ⅱ multiplex ssLAMP assay with BA.1 plasmid and BA.3 plasmid. B) system Ⅱ multiplex LAMP assay with BA.1 plasmid and BA.3 plasmid.


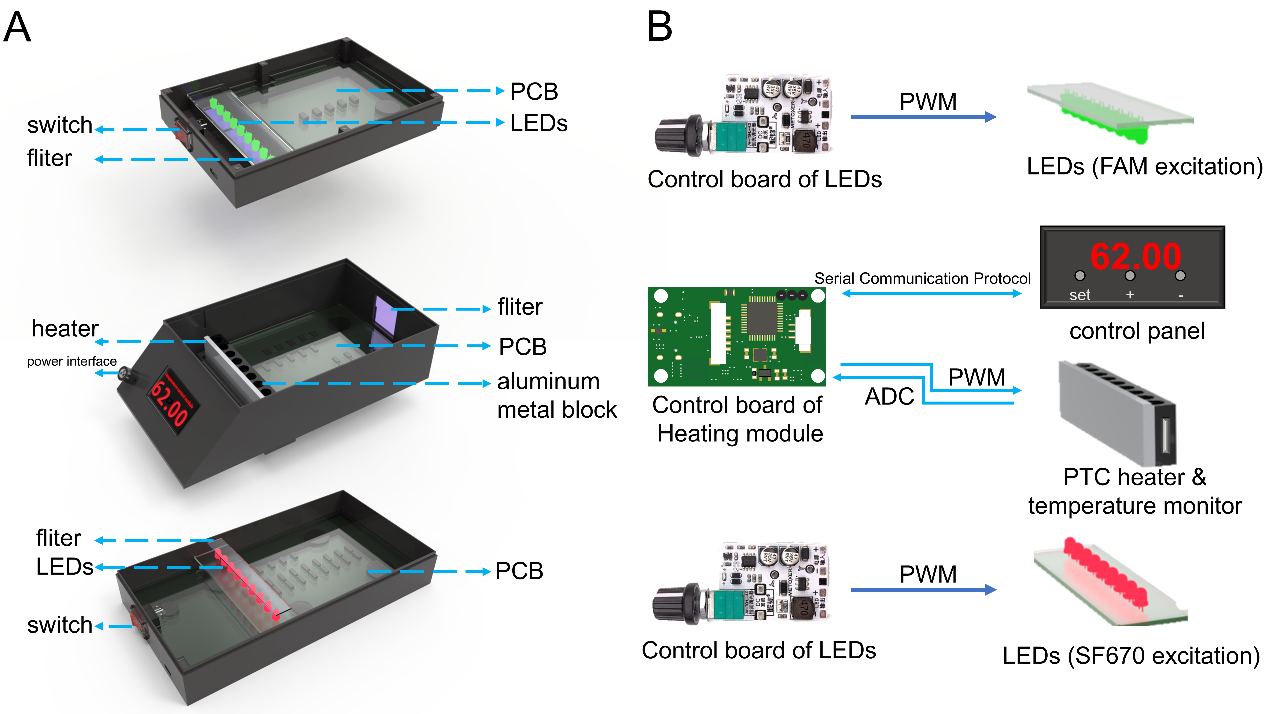


**Figure S7. The overall design of the portable testing device.** A. The 3D module exploded view of the device, which is composed of two excitation light module and a heating module. B. PCB architecture diagram of the control system.


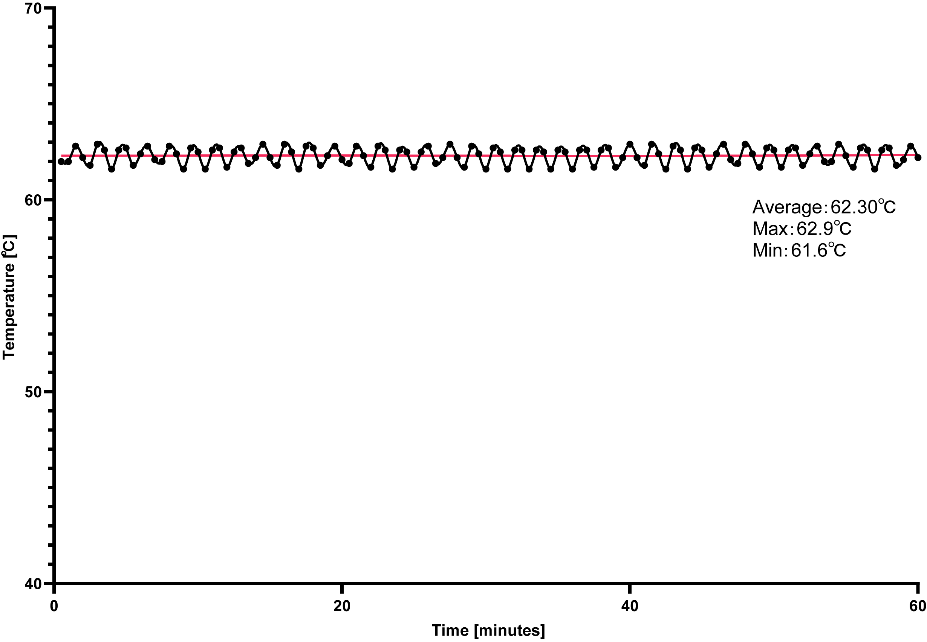


**Figure S8. Temperature changes in the portable device’s heating module within 60 minutes.**


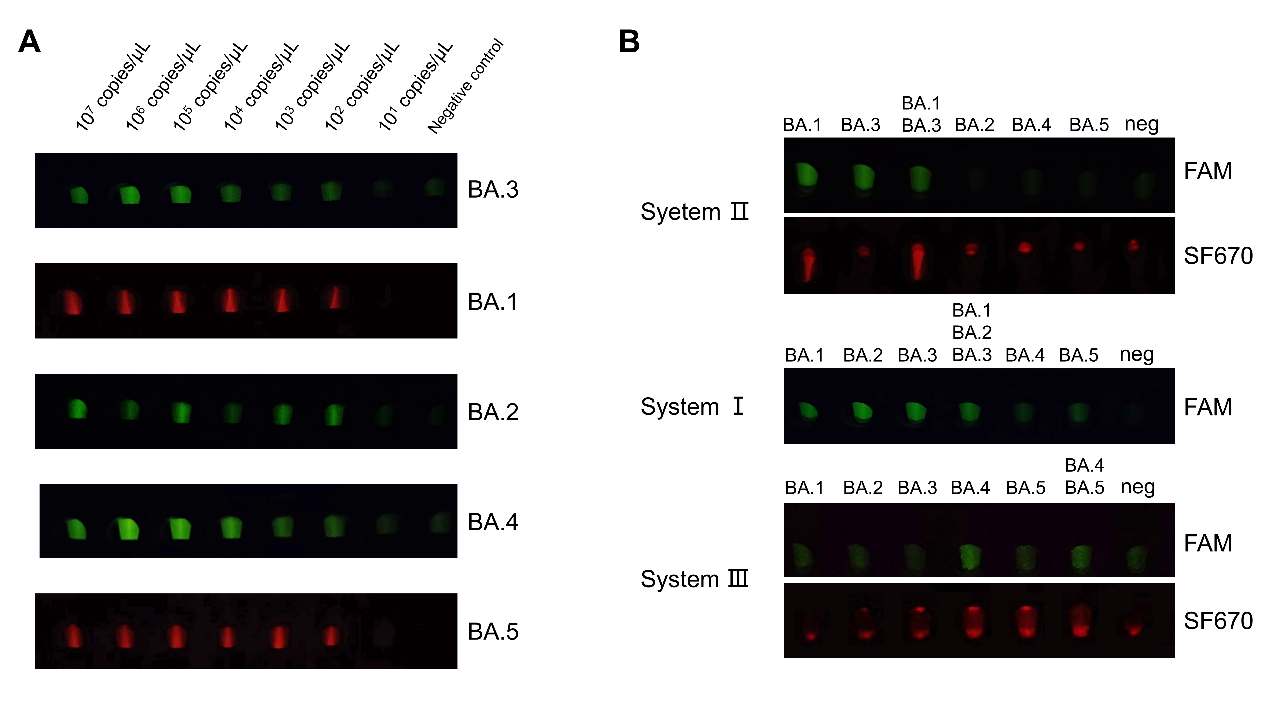


**Figure S9. The sensitivity and specificity test of ssLAMP** **when utilising the portable device.** A) The sensitivity test using different plasmid templates with gradient concentration. B) The specificity test of different detection systems of the ssLAMP for SARS-CoV-2 detection.


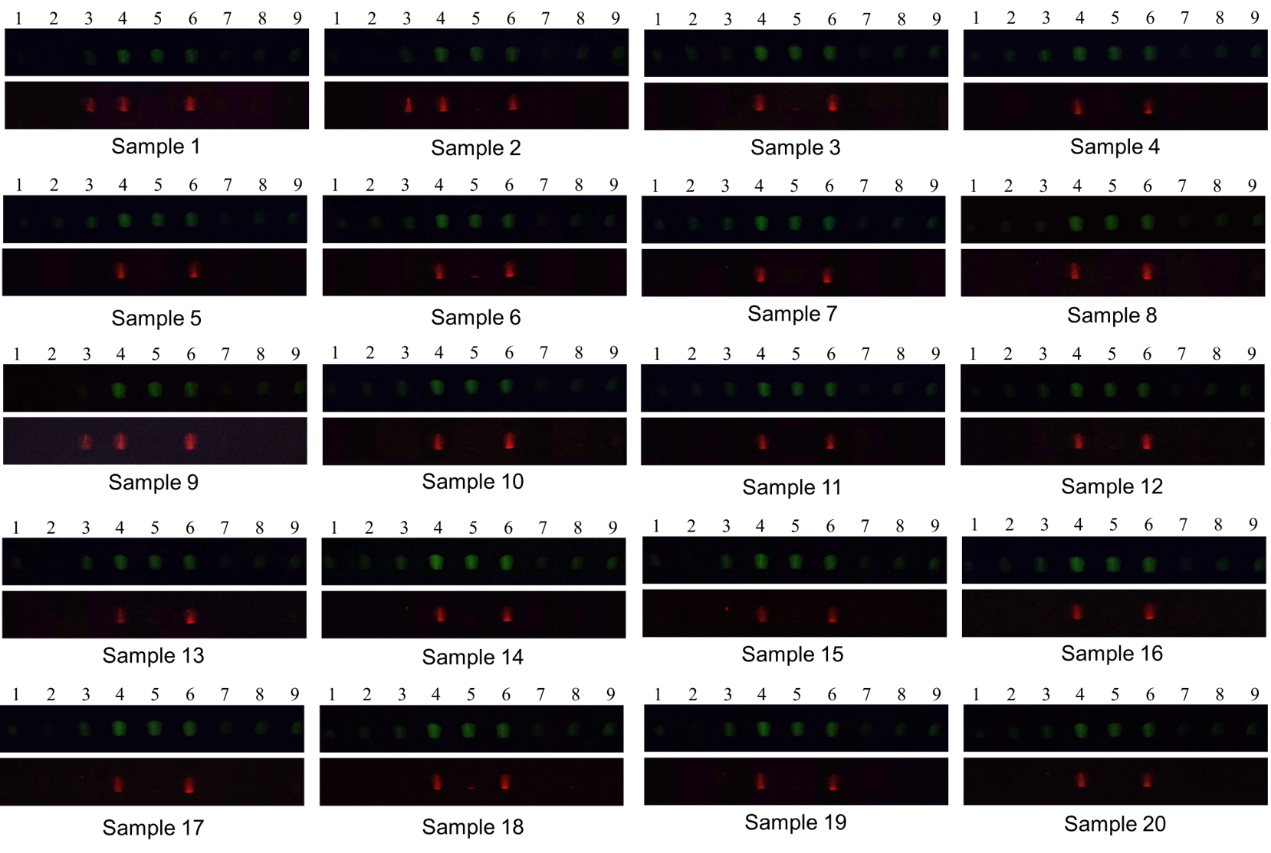


**Figure S10. Testing result of sample 1-20 using ssLAMP assays.**


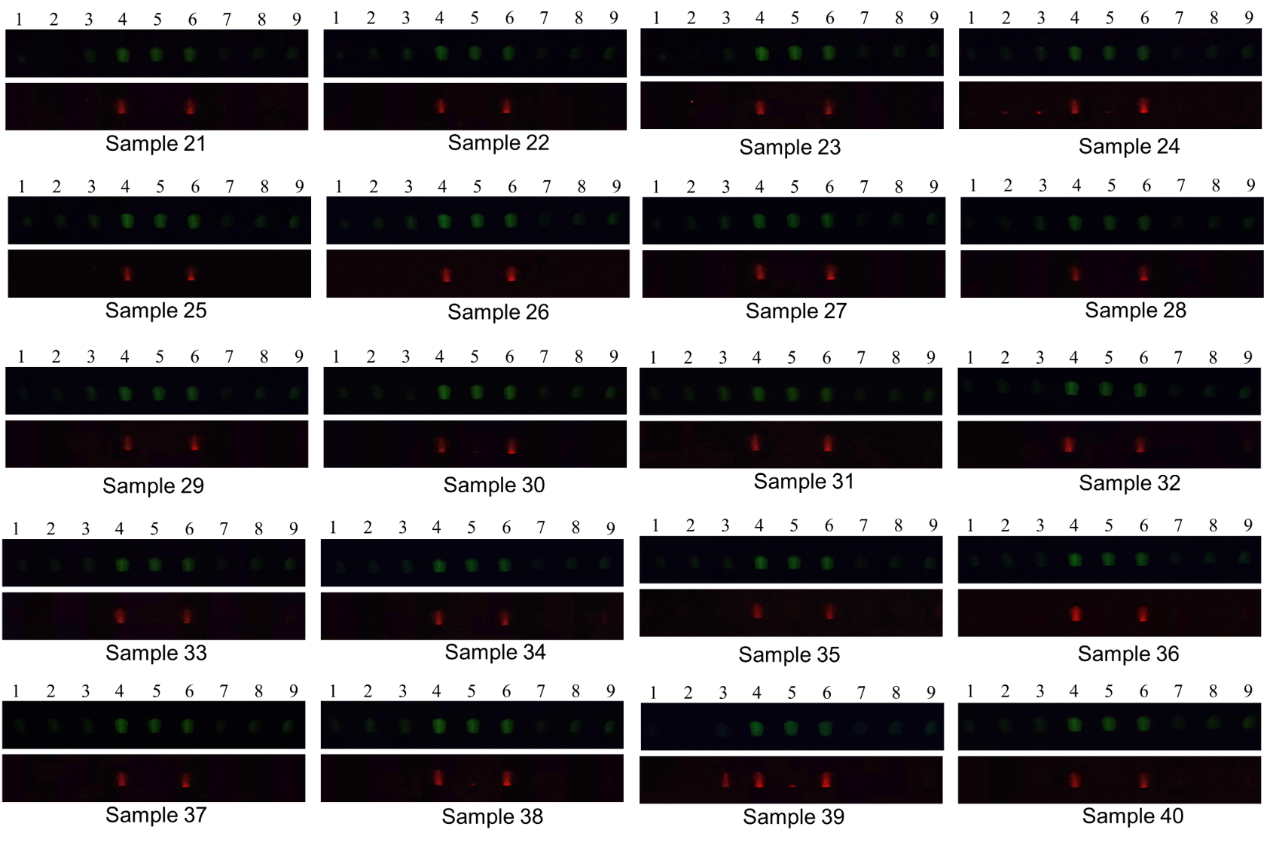


**Figure S11. Testing result of sample 21-40 using ssLAMP assays.**


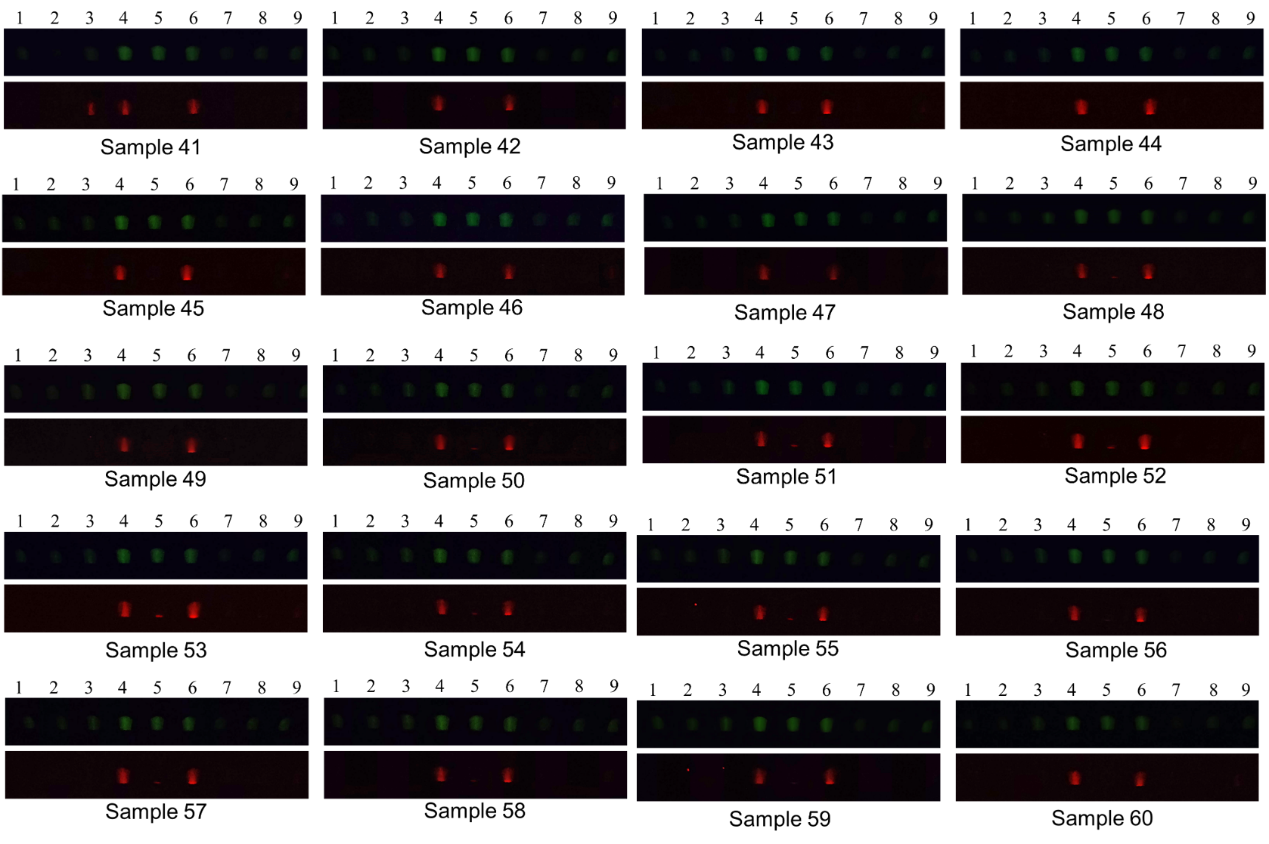


**Figure S12. Testing result of sample 41-60 using ssLAMP assays.**


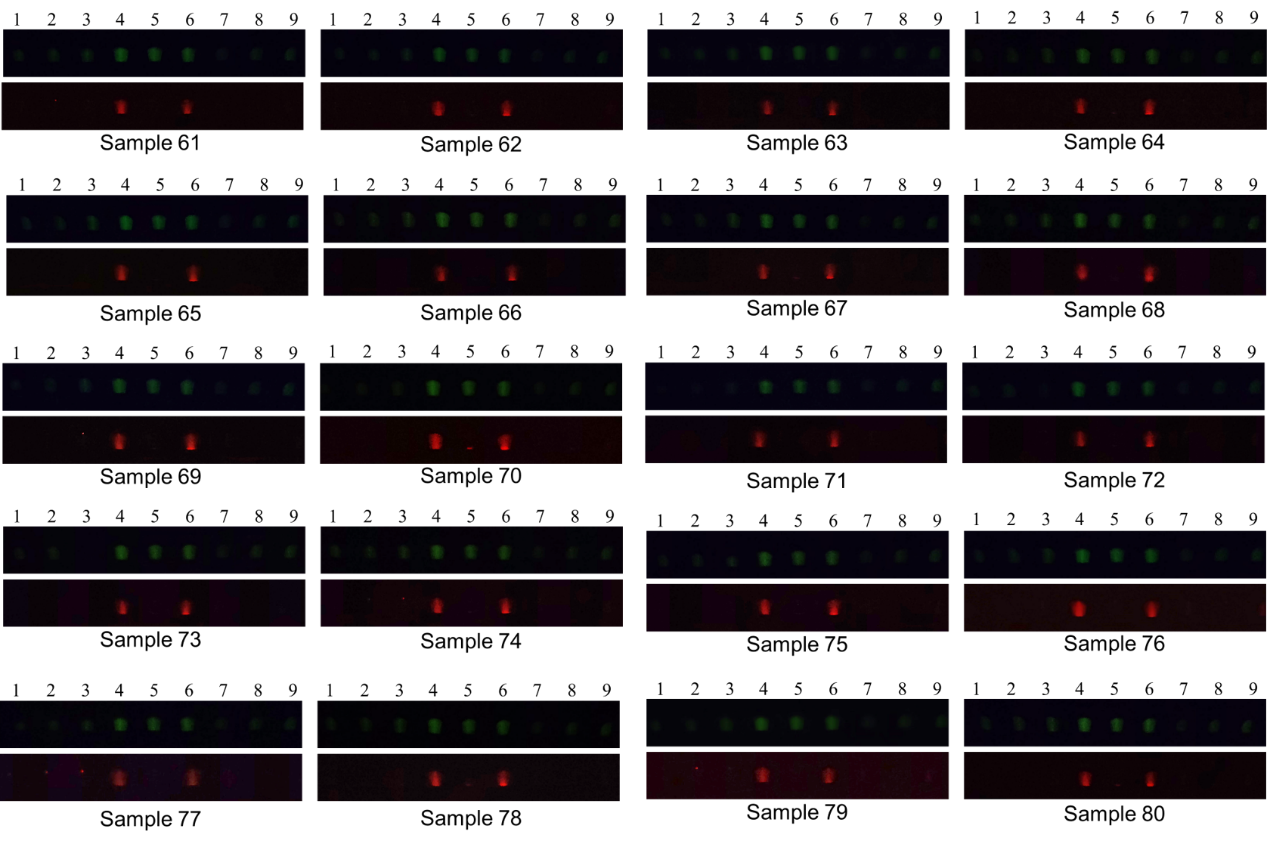


**Figure S13. Testing result of sample 61-80 using ssLAMP assays.**


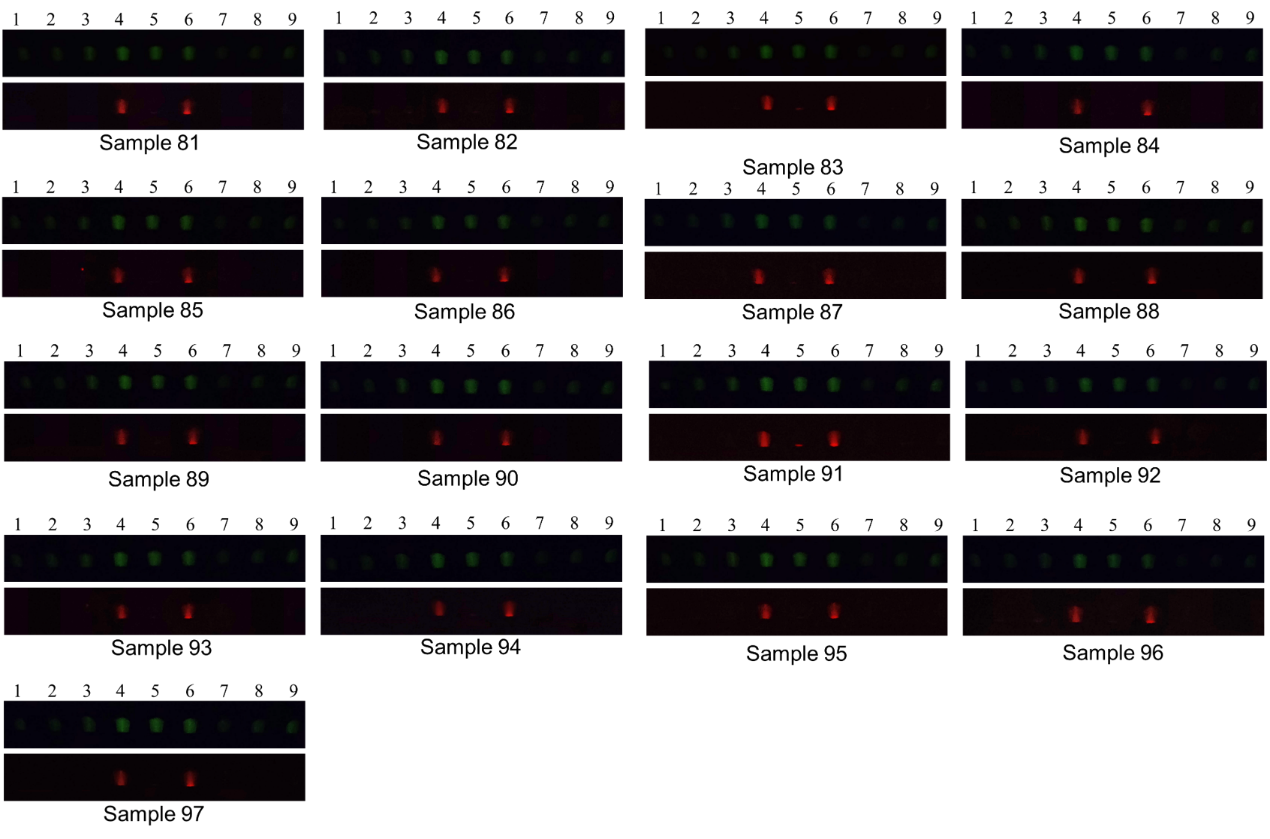


**Figure S14. Testing result of sample 81-97 using ssLAMP assays.**


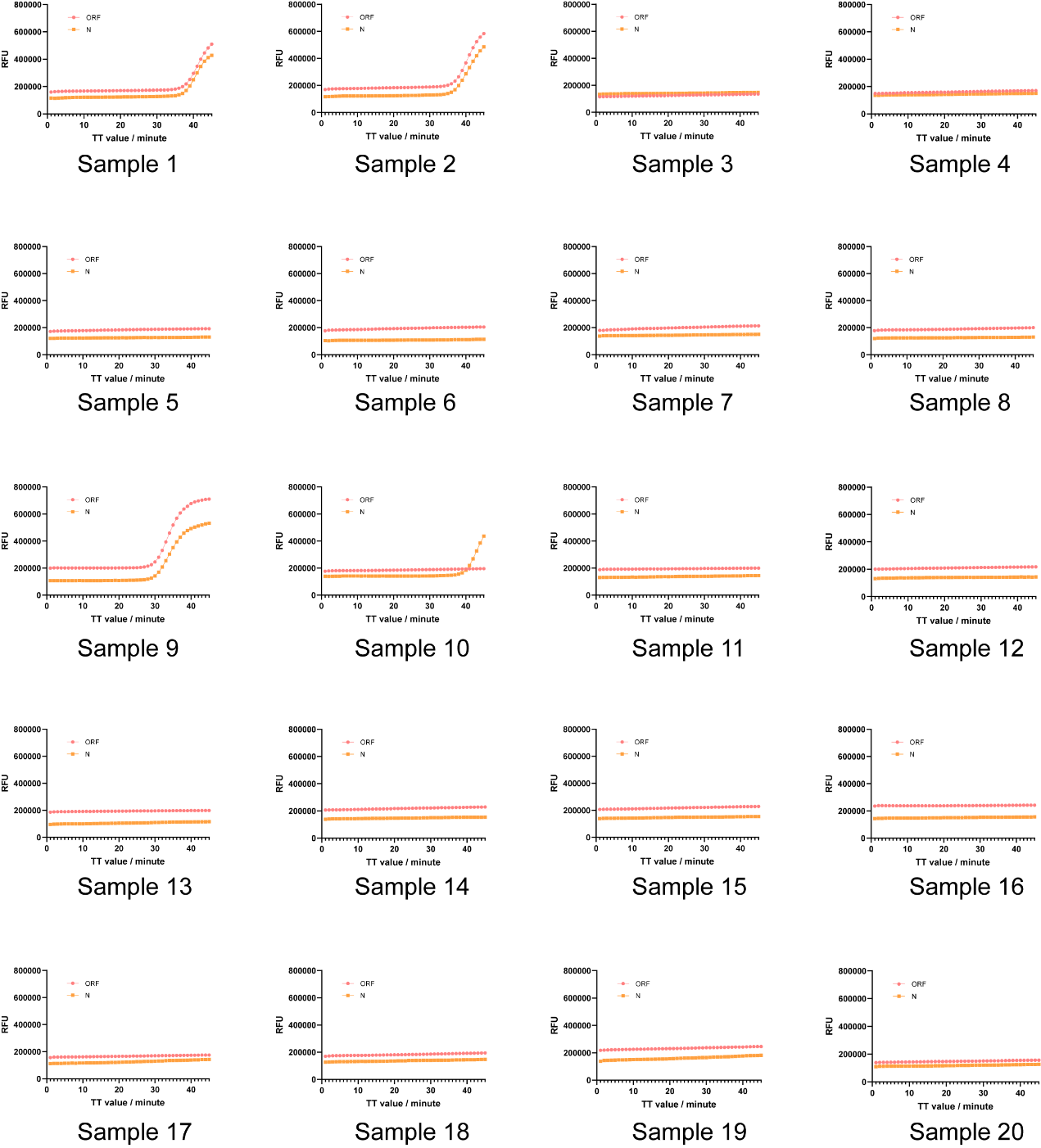


**Figure S15. Testing result of sample 1-20 using qPCR assays.**


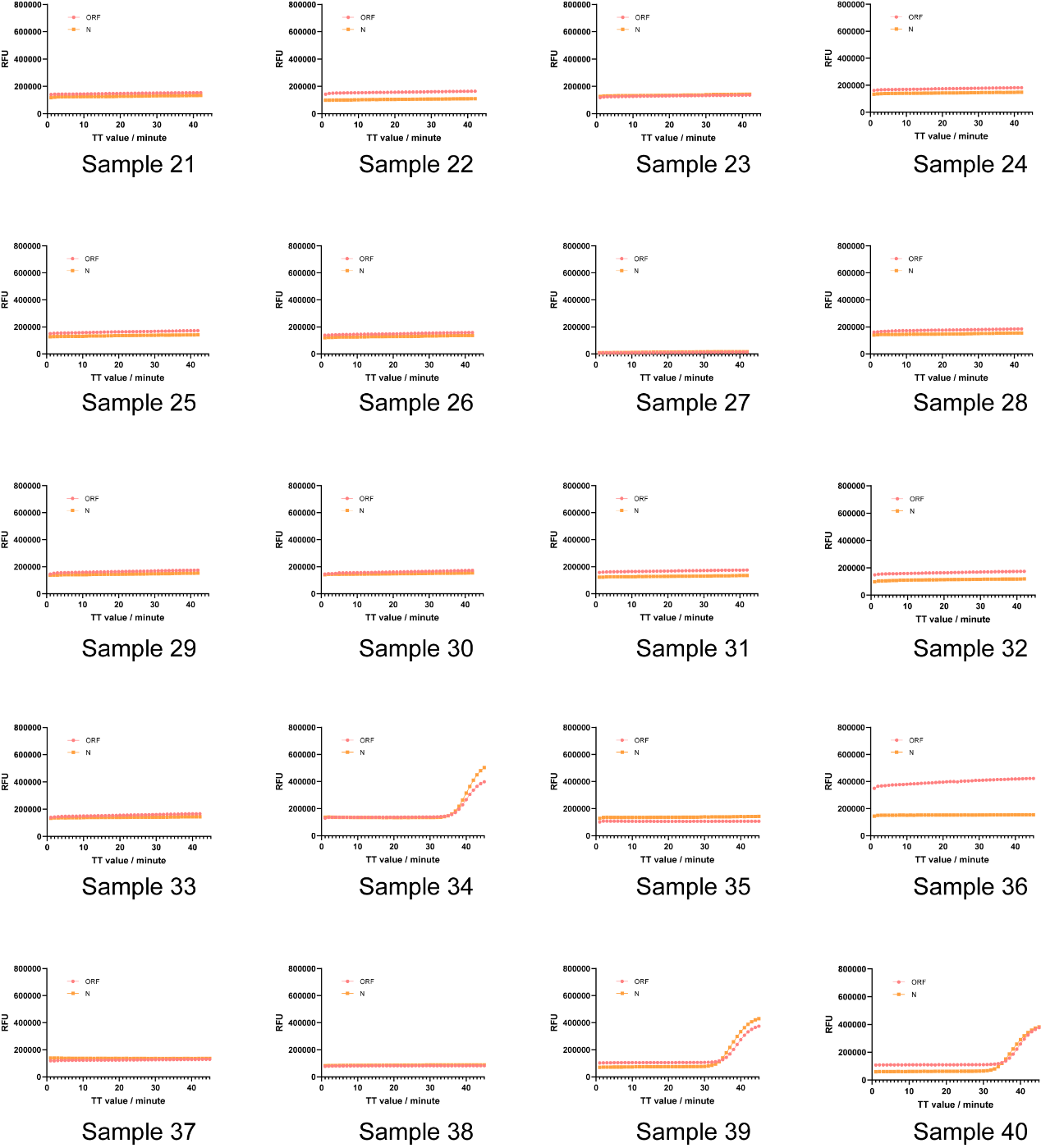


**Figure S16. Testing result of sample 21-40 using qPCR assays.**


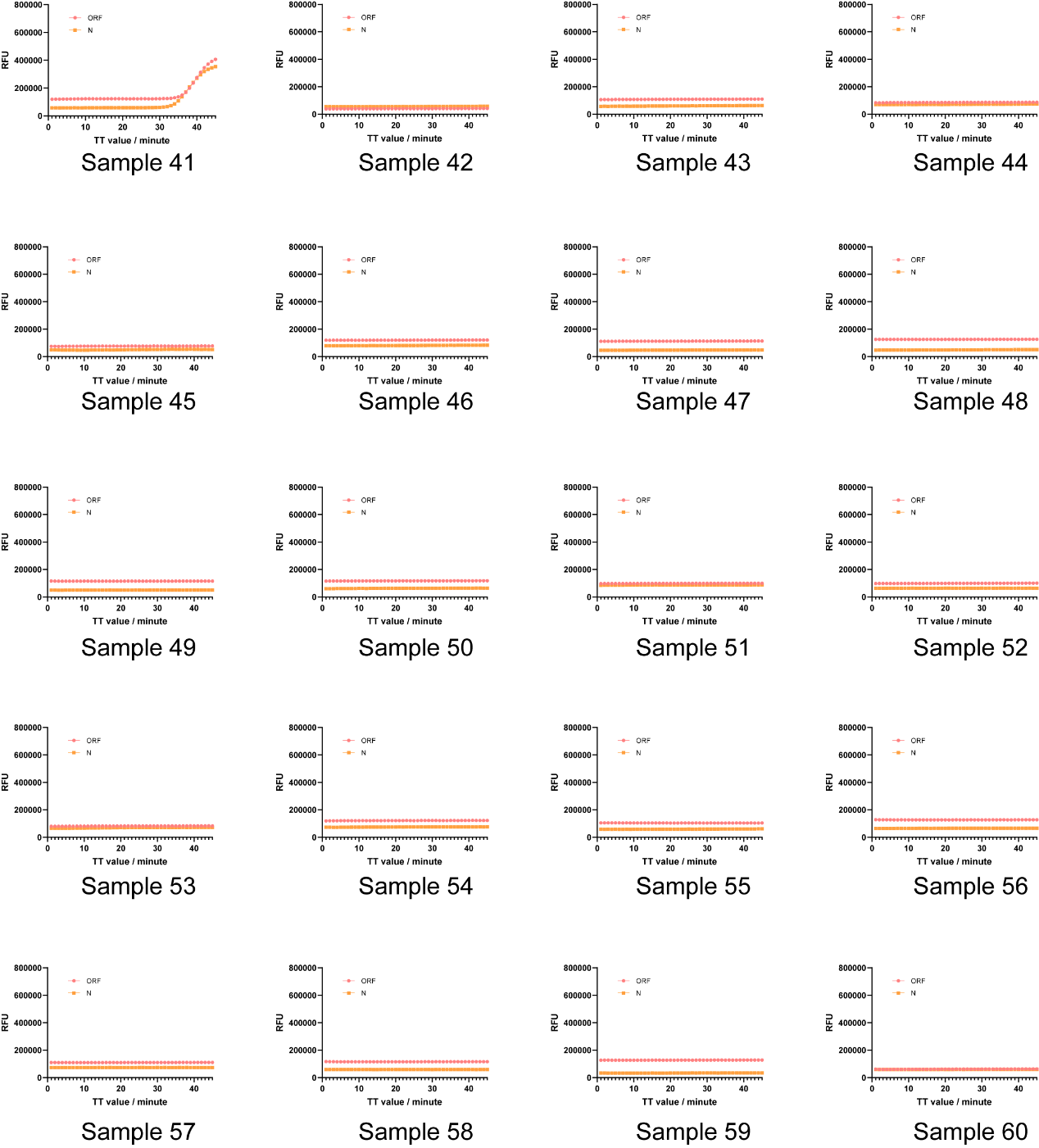


**Figure S17. Testing result of sample 41-60 using qPCR assays.**


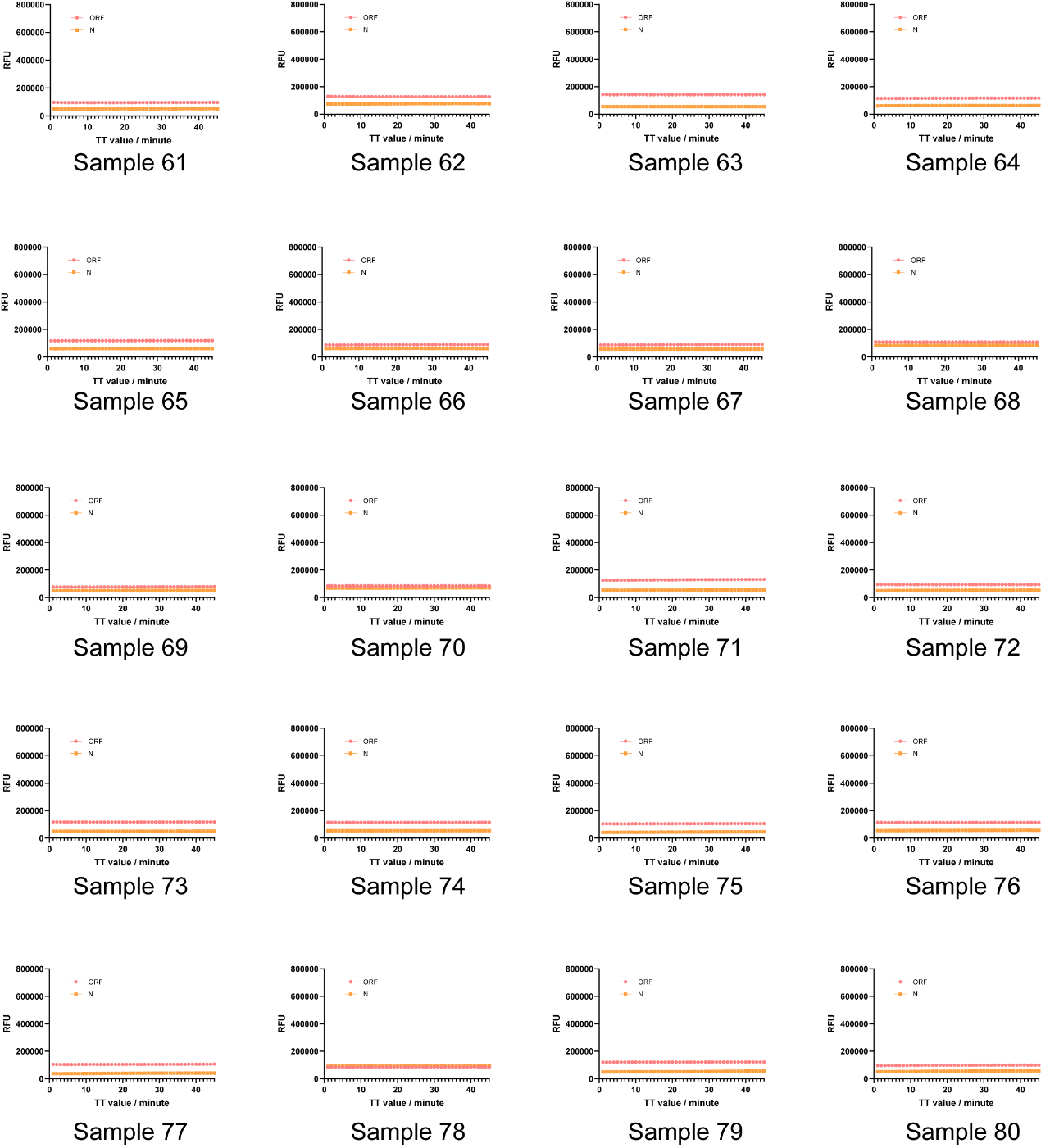


**Figure S18. Testing result of sample 61-80 using qPCR assays.**


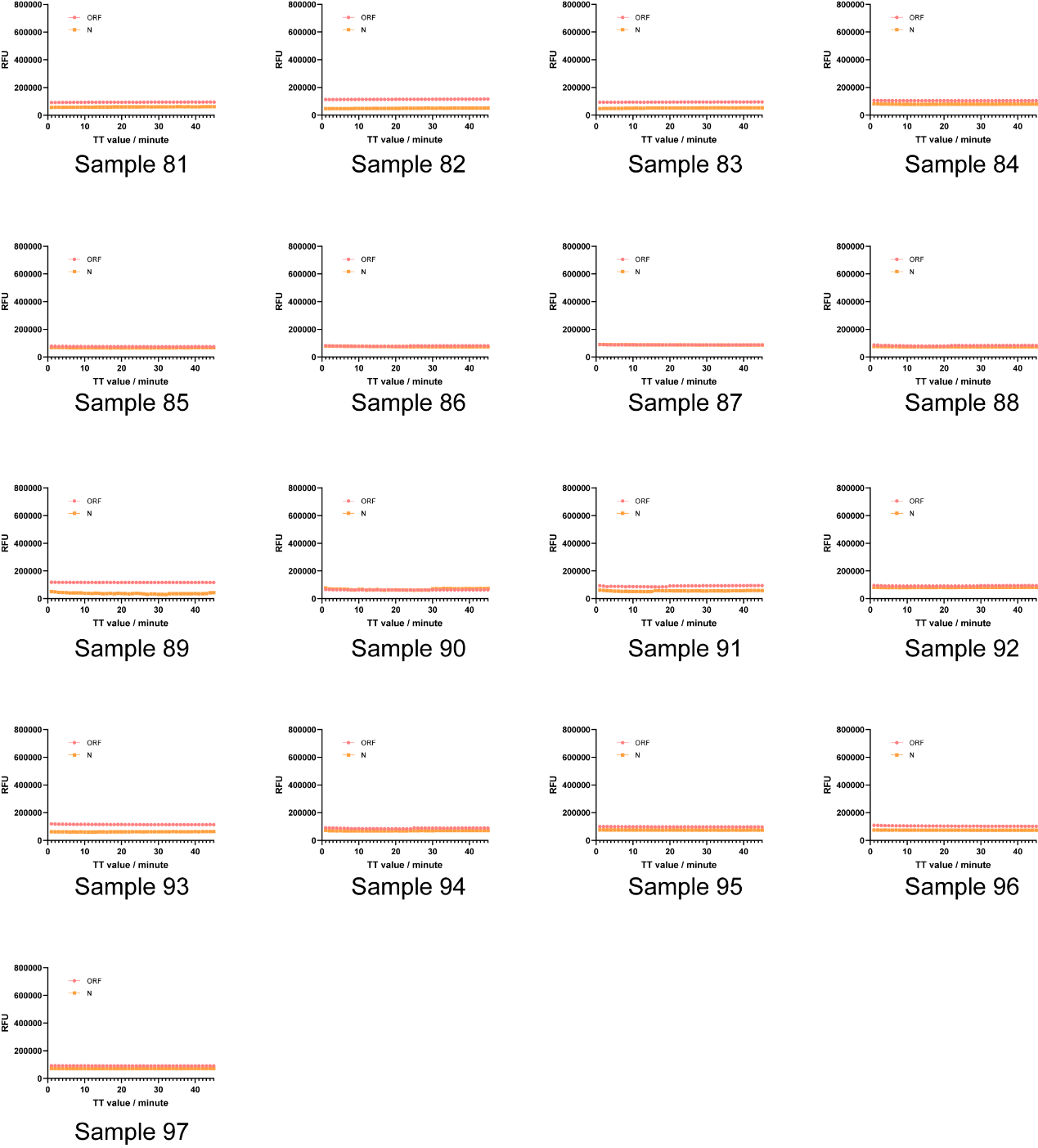


**Figure S19. Testing result of sample 81-97 using qPCR assays.**

**Figure S20. The Sanger sequencing results of clinical samples identified as SARS-CoV-2 Omicron positive.**


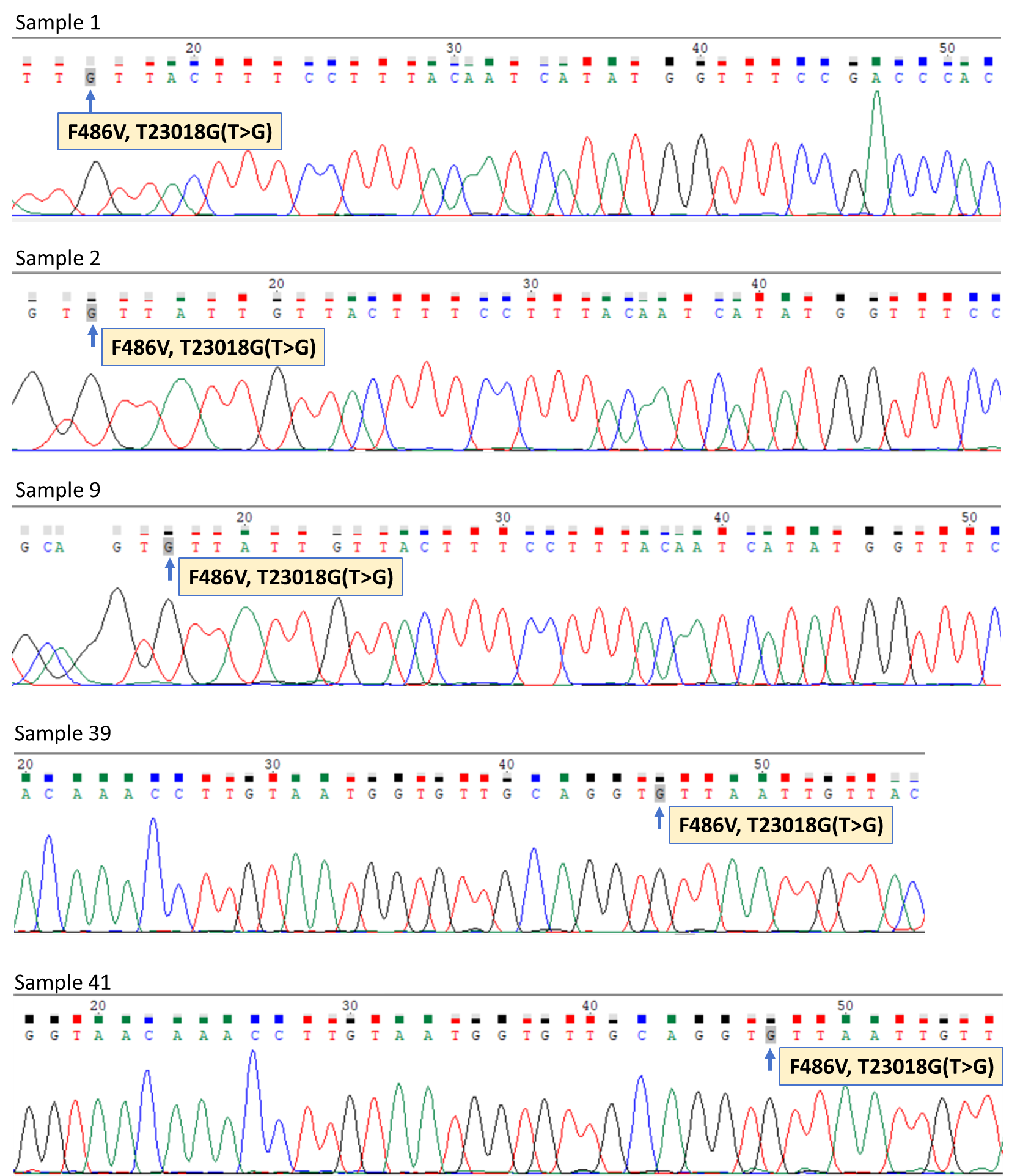

Supplement: Supplementary file 1 — Supporting Information [file ADVS-12-2502708-s001.docx]
